# Delineating transitions during the evolution of specialised peroxisomes: Glycosome formation in kinetoplastid and diplonemid protists

**DOI:** 10.3389/fcell.2022.979269

**Published:** 2022-09-12

**Authors:** Diego Andrade-Alviárez, Alejandro D. Bonive-Boscan, Ana J. Cáceres, Wilfredo Quiñones, Melisa Gualdrón-López, Michael L. Ginger, Paul A. M. Michels

**Affiliations:** ^1^ Laboratorio de Enzimología de Parásitos, Departamento de Biología, Facultad de Ciencias, Universidad de Los Andes, Mérida, Venezuela; ^2^ School of Public Health, University of Alberta, Edmonton, AB, Canada; ^3^ School of Applied Sciences, University of Huddersfield, Huddersfield, United Kingdom; ^4^ Centre for Immunity, Infection and Evolution and Centre for Translational and Chemical Biology, School of Biological Sciences, The University of Edinburgh, Edinburgh, United Kingdom

**Keywords:** Euglenozoa, peroxisome, phylogenomics, metabolic compartmentalisation, biogenesis, pexophagy, glycosome, glycolysis

## Abstract

One peculiarity of protists belonging to classes Kinetoplastea and Diplonemea within the phylum Euglenozoa is compartmentalisation of most glycolytic enzymes within peroxisomes that are hence called glycosomes. This pathway is not sequestered in peroxisomes of the third Euglenozoan class, Euglenida. Previous analysis of well-studied kinetoplastids, the ‘TriTryps’ parasites *Trypanosoma brucei*, *Trypanosoma cruzi* and *Leishmania* spp., identified within glycosomes other metabolic processes usually not present in peroxisomes. In addition, trypanosomatid peroxins, *i.e.* proteins involved in biogenesis of these organelles, are divergent from human and yeast orthologues. In recent years, genomes, transcriptomes and proteomes for a variety of euglenozoans have become available. Here, we track the possible evolution of glycosomes by querying these databases, as well as the genome *of Naegleria gruberi*, a non-euglenozoan, which belongs to the same protist supergroup Discoba. We searched for orthologues of TriTryps proteins involved in glycosomal metabolism and biogenesis. Predicted cellular location(s) of each metabolic enzyme identified was inferred from presence or absence of peroxisomal-targeting signals. Combined with a survey of relevant literature, we refine extensively our previously postulated hypothesis about glycosome evolution. The data agree glycolysis was compartmentalised in a common ancestor of the kinetoplastids and diplonemids, yet additionally indicates most other processes found in glycosomes of extant trypanosomatids, but not in peroxisomes of other eukaryotes were either sequestered in this ancestor or shortly after separation of the two lineages. In contrast, peroxin divergence is evident in all euglenozoans. Following their gain of pathway complexity, subsequent evolution of peroxisome/glycosome function is complex. We hypothesize compartmentalisation in glycosomes of glycolytic enzymes, their cofactors and subsequently other metabolic enzymes provided selective advantage to kinetoplastids and diplonemids during their evolution in changing marine environments. We contend two specific properties derived from the ancestral peroxisomes were key: existence of nonselective pores for small solutes and the possibility of high turnover by pexophagy. Critically, such pores and pexophagy are characterised in extant trypanosomatids. Increasing amenability of free-living kinetoplastids and recently isolated diplonemids to experimental study means our hypothesis and interpretation of bioinformatic data are suited to experimental interrogation.

## Introduction

**FIGURE 1:**
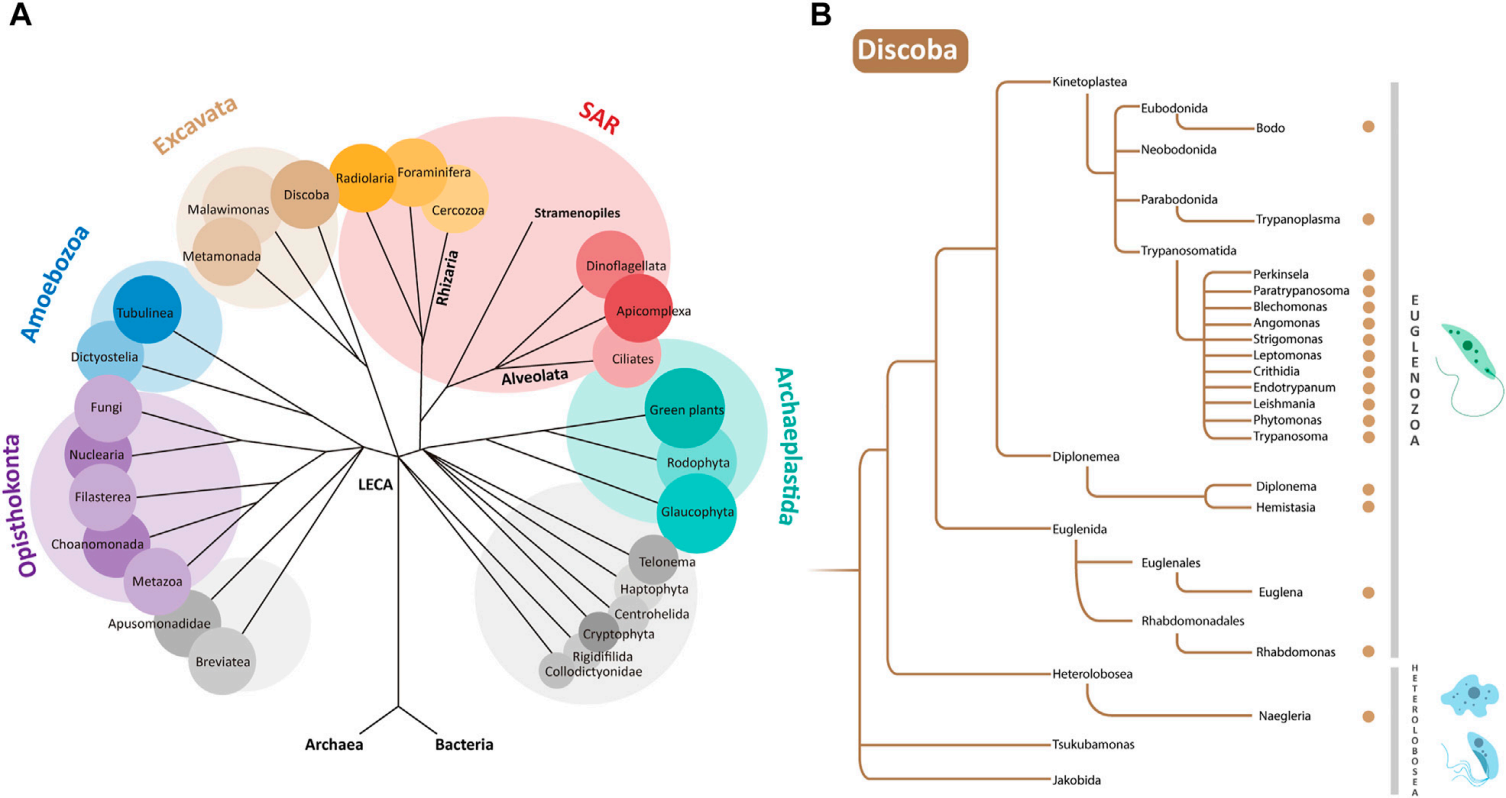
**(A)** Phylogenetic relationships of eukaryotes. LECA is the “Last Eukaryotic Common Ancestor” from which different supergroups evolved. Euglenozoa are a phylum within the supergroup Discoba belonging to the Excavata (Adl et al., 2019). **(B)** Zoom of a simplified representation of the Discoba clade. Genera containing organisms discussed in this paper are indicated by brown dots. Examples of organisms from the Euglenozoa and Heterolobosea phyla are depicted at the right.

Glycosomes are peroxisomes that compartmentalise glycolytic enzymes. These organelles were initially detected in the African sleeping sickness parasite *Trypanosoma brucei* as single-membrane bounded microbodies containing glycolytic enzymes, hence called glycosomes (Opperdoes and Borst, 1977). Glycosomes were subsequently observed in other members of the parasitic taxonomic order Trypanosomatida, class Kinetoplastea, including *Trypanosoma cruzi* and *Leishmania* spp, and then found in bodonid representatives of the Kinetoplastea (Figure 1) (Taylor et al., 1980; Hart and Opperdoes, 1984; Opperdoes et al., 1988; Ardelli et al., 2000). The range of enzymes detected in glycosomes also broadened; notably, from the perspective of carbohydrate metabolism key enzymes of gluconeogenesis, phosphoenolpyruvate carboxykinase (PEPCK) and fructose-1,6-bisphosphatase (FBPase), were found to be glycosomal (Parsons and Smith, 1989; Hannaert et al., 2003a).

Trypanosomatid protists are relatively well known as some species are aetiological agents of serious neglected tropical diseases in people, veterinary diseases, or are plant pathogens (Jaskowska et al., 2015; Giordani et al., 2016; Field et al., 2017). With rare exception, these parasites are transmitted between hosts by an insect vector. Indeed, the vast majority of trypanosomatids are simply monoxenous parasites of insects. Yet, irrespective of whether trypanosomatids are monoxenous or dixenous in lifestyle, their life cycles are invariably complex requiring differentiation through different morphological forms, and often adaptation of their metabolic network to different nutritional conditions (*e.g.* Wheeler et al., 2013; Atwood et al., 2005; Urbaniak et al., 2012). Such adaptation includes enzyme content of the glycosomes, with important changes in the expression of glycolytic enzymes. *T. brucei* provides a prime example. Here, parasites residing in the mammalian bloodstream require glycolysis for ATP generation and glycolytic enzymes comprise 90–95% of the glycosomal protein content. In contrast, in the digestive tract of the tsetse fly vector sugar availability is typically low and pathways other than glycolysis are preferred for ATP production (Michels et al., 2021); although glycosomes are now used for gluconeogenesis (Wargnies et al., 2018), levels of glycosomal glycolytic enzymes are 40–50% that of bloodstream trypanosomes (Hart et al., 1984; Misset et al., 1986).

Enzymes from other ubiquitous metabolic systems that are generally not present or found rarely in peroxisomes of mammals, plants, or yeasts have also been fully or partially localised to trypanosomatid glycosomes. The diverse list includes some enzymes of pyrimidine synthesis, purine salvage and squalene biosynthesis, many of the enzymes participating in nucleotide-sugar biosynthesis, some additional enzymes of carbohydrate metabolism such as those involved in the pentose-phosphate pathway (PPP), succinic fermentation and gluconeogenesis (Haanstra et al., 2016; Allmann and Bringaud, 2017; Quiñones et al., 2020; Michels et al., 2021). Initially, these enzymes were reported as glycosomal based on cell fractionation and enzyme assays in one or several different trypanosomatids (e.g., Hammond et al., 1981; Hammond and Gutteridge, 1982; Concepción et al., 1998; Heise and Opperdoes, 1999; Hannaert et al., 2003a; Coustou et al., 2006; Sampaio Guther et al., 2021). Latterly, these reports were confirmed or extended by mass spectrometry-based proteomics studies (Colascante et al., 2006; Vertommen et al., 2008; Colasante et al., 2013; Güther et al., 2014; Acosta et al., 2019).

Despite presence of a single boundary membrane, high protein content and no nucleic acids, unusual enzyme content coupled to an absence of catalase led initially to scepticism about assignment of glycosomes to the peroxisome family. However, indication of the evolutionary relationship of glycosomes with peroxisomes came first from detection of ‘classic’ peroxisome enzymes, such as those involved in ether-lipid biosynthesis (Opperdoes, 1984) or fatty-acid β-oxidation (Hart and Opperdoes, 1984; Wiemer et al., 1996), in trypanosome glycosomes. Subsequently, identification of protein targeting to glycosomes using typical peroxisomal-targeting signals (PTS) – a C-terminal tripeptide (PTS1) or a nonapeptide near the N-terminus (PTS2) – and the requirement of peroxin proteins for organelle biogenesis showed unequivocally that glycosomes are specialized peroxisomes, unique for their sequestration of many enzymes required for glycolysis or the extended metabolism of glycolytic intermediates (reviewed in Gualdrón-López et al., 2012a; Gabaldón et al., 2016). Quite why and how a kinetoplastid ancestor remodelled peroxisome function so dramatically remain open questions in evolutionary biology. Here, we address these questions through analysis of recently available genomes and transcriptomes from across the Euglenozoa, the phylum to which kinetoplastids belong, and survey of the wider literature. From the data, we provide a fresh view of the cumulative gain of glycosomal metabolic complexity during euglenozoan evolution and offer an original posit that divergence evident in trypanosomatid peroxins occurred early during euglenozoan evolution.

### Identification of candidate glycosomal proteins in Euglenozoa

The classes Kinetoplastea, Diplonemea, and Euglenida provide the three major lineages of the Euglenozoa (Kostygov et al., 2021). There is also an enigmatic fourth lineage, Symbiontida (Yubuki and Leander, 2018) but in the absence of genome or transcriptome data, these little-studied euglenozoans are not considered further here. Instead, to trace the origin of glycosomes from peroxisomes and their subsequent evolution within **Euglenozoa**, taxa were selected as listed below. From **Kinetoplastea**: *Trypanosoma brucei, Trypanosoma vivax, Trypanosoma cruzi, Leishmania major, Endotrypanum monterogeii* (all dixenous–*i.e*., having two hosts–parasites of mammals, transmitted by insect vectors), *Leishmania tarentolae* (a reptile parasite), *Crithidia fasciculata, Blechomonas ayalai, Leptomonas pyrrhocoris, Leptomonas seymouri, Paratrypanosoma confusum, Angomonas deanei, Strigomonas culicis* (all monoxenous insect parasites), *Phytomonas* (a dixenous plant parasite), *Perkinsela* sp. (an endosymbiont of the amoeba *Paramoeba*), *Trypanoplasma borreli* (a fish parasite) and *Bodo saltans* (a free-living flagellate). From **Diplonemea**: *Diplonema papillatum^1^, Diplonema japonicum, Diplonema ambulator, and Hemistasia phaeocysticola* (all free-living organisms). From **Euglenida**: *Euglena gracilis, Euglena longa* and *Rhabdomonas costata* (all free-living organisms).

Euglenozoa belong to the protist supergroup **Discoba** (Adl et al., 2019; Burki et al., 2020), which also contains the phylum **Heterolobosea.** For use as a not-distant outgroup in our analysis, we selected from Heterolobosea species of *Naegleria* known to contain peroxisomes (González-Robles et al., 2020): *N. gruberi* (a free-living soil and freshwater amoeboflagellate), *N. fowleri* (a free-living, but deadly opportunistic pathogen) and *N. lovaniensis* (a free-living thermophile). Phylogenetic relationships between all organisms listed are summarised in Figure 1B.

In searches, amino-acid sequences of known glycosomal proteins from different organisms provided queries to retrieve orthologues and paralogues from the following databases: TriTrypDB (https://tritrypdb.org/tritrypdb/app), AmoebaDB (https://amoebadb.org/amoeba/app), OrthoMCL DB (https://orthomcl.org/orthomcl/app), NCBI (National Center for Biotechnology Information, https://www.ncbi.nlm.nih.gov/) and DiscobaDB (https://zenodo.org/record/5563074) (Wheeler, 2021). Query sequences were adopted on a basis of glycosomal localisation from proteomic and genomic studies (Colasante et al., 2006, 2013; Güther et al., 2014; Morales et al., 2016; Acosta et al., 2019; Durrani et al., 2020; González-Robles et al., 2020; Quiñones et al., 2020; Jansen et al., 2021; Wheeler, 2021; Škodová-Sveráková et al., 2021) or the many biochemical and cell biological studies published previously. Sequence screening in DiscobaDB (Wheeler, 2021) was done using Geneious Prime^®^ 2022.0.1 and BLASTp with default parameters. OrthoMCL DB was used to verify the number of orthologues or target genes during data collection in TriTrypDB and AmoebaDB.

Amino-acid sequences retrieved were analysed considering specific criteria: the presence of PTS2 and/or PTS1 motifs, and possible experimental evidence of their localisation in glycosomes or peroxisomes. PTS signatures were identified using the ScanProsite Tool (https://prosite.expasy.org/scanprosite/; option 3: “Submit PROTEIN sequences and MOTIFS to scan them against each other”) with the following motifs for **PTS1** (**A**) [ASCGPNYTV]-[KNRHQDS]-[LMVAIF]> (Acosta et al., 2019) (**B**) [STAGCN]-[RKH]-[LIVMAFY]> (**C**) S-S-[LIF]> (Opperdoes and Skizora, 2006) (**D**) [ACGHNPT]-[HMNQRS]-[IMY]> (Sommer et al., 1992); and for **PTS2** (**E**) [RKHQ]-[VLIWFY]-X (5)-[HKQR]-[ILVYAF] (Acosta et al., 2019) (**F**) <M-X(0,20)-[RK]-[LVI]-X(5)-[HQ]-[ILAF] (Opperdoes and Skizora, 2006) (**G**) R-[LVIQ]-X(2)-[LVIH]-[LSGA]-X-[HQ]-[LA] (**H**) [RK]-[LVIQ]-X(2)-[LVIHQ]-[LSGAK]-X-[HQ]-[LAF] (Petriv et al., 2004) and (**I**) R-[LI]-X(2)-[LI]-X (2)-[HQ]-L (Kunze, 2020). This analysis was applied for all orthologues or paralogues of metabolic enzymes considered to be possibly present inside glycosomes/peroxisomes, except for candidate biogenesis proteins (peroxins). For the latter, the screening for specific domains was applied using the web server HMMER (https://www.ebi.ac.uk/Tools/hmmer/search/phmmer) and InterProScan (https://www.ebi.ac.uk/interpro/result/InterProScan/). Peroxin sequence alignments were made using MAFFT (https://www.ebi.ac.uk/Tools/msa/mafft/) and MUSCLE (https://www.ebi.ac.uk/Tools/msa/muscle/) and visualized in BioEdit Sequence Alignment Editor, v7.2.5.

### Under what condition(s) did glycosomes originate?

Long-standing questions about glycosomes are when, in an ancestral organism, peroxisomes acquired novel functions to become glycosomes; when did they lose some typical peroxisomal functions such as part of the H_2_O_2_-dependent metabolism; how did it happen and what could have been the selective advantage(s) (Borst, 1986; Opperdoes, 1987; Borst, 1989; Michels and Opperdoes, 1991; Michels and Hannaert, 1994; Hannaert et al., 2003b; Gualdrón-López et al., 2012a; Gabaldón et al., 2016)? A possible evolutionary scenario was described in Gualdrón-López et al. (2012a). However, this scenario was based on limited knowledge then available: the analysis of the ‘TriTryp’ (*T. brucei*, *T. cruzi* and *L. major*) genomes (El-Sayed et al., 2005); from the biochemical characterisation of glycosomes from several trypanosomatids and the parasitic bodonid *T. borreli* (Opperdoes et al., 1988); and discovery of a PTS2 motif in the glycolytic enzyme aldolase from the diplonemid *D. papillatum* coupled to its localisation in cell compartments reminiscent of glycosomes (Makuichi et al., 2011). The presence of glycosomes was subsequently confirmed in *D. papillatum* (Morales et al., 2016), indicating glycosome origin predated divergence of a last common kinetoplastid ancestor. However, biochemical analyses of carbohydrate metabolism suggests *D. papillatum* glycosomes principally function in the direction of gluconeogenesis, rather than glycolysis (Morales et al., 2016; Škodová-Sveráková et al., 2021). In *D. papillatum*, a marine flagellate, amino acids are considered to provide the predominant carbon source for central energy metabolism. Detection of organellar FBPase activity but no phosphofructokinase (PFK) supported an idea that ancestrally gluconeogenesis provided a selective driver for glycosome evolution (Morales et al., 2016). From transcriptome data it is now known that *D. papillatum* expresses a PFK that has homology to pyrophosphate (PPi)-dependent PFKs from other organisms and contains a PTS1 motif (Škodová-Sveráková et al., 2021).

Critically, realisation that the appearance of glycosomes predates a last common ancestor of diplonemids and kinetoplastids argues we need to look widely across the Euglenozoa for when the metabolic functionality of their peroxisomes began to diversify. *Euglena gracilis*, in which peroxisomes lack glycolytic enzyme activities (Opperdoes et al., 1988) provides a poor proxy for understanding peroxisome diversity among euglenids. There are ∼1,000 euglenid species known and photosynthetic euglenids represent only one subclade within the group (Lax et al., 2021). Most euglenids are free-living heterotrophic phagotrophs; some are non-photosynthetic osmotrophs. With availability of a *R. costata* genome sequence it becomes possible to begin to look more widely among euglenids at peroxisome diversity.

Euglenids, diplonemids, and kinetoplastids are all cosmopolitan with respect to their geographic distributions. Euglenids and free-living kinetoplastids are ubiquitous in moist soils and species from all three lineages are readily found in both freshwater and marine environments. Indeed, ribosomal DNA sequencing of planktonic samples recently and unexpectedly revealed Diplonemea as an extremely diverse, abundant class of flagellates throughout temperate and tropical oceans. They appear particularly abundant in the nutrient-poor, colder mesopelagic zone of the oceans (*i.e.*, approximately 200 – 1,000 m below the ocean surface) and are also present at abyssal and hadal depths. Kinetoplastids are also found in a similarly diverse array of oceanic niches (Salani et al., 2012; Flegontova et al., 2020; Kostygov et al., 2021; Schoenle et al., 2021).

If it was in a common ancestor of Diplonemea and Kinetoplastea in which glycolysis became uniquely compartmentalised within peroxisomes, then it is reasonable to estimate that this ancestor lived >600 million years ago, most likely in the oceans, at a point in Earth’s history when only the uppermost regions of the oceans were considered oxygenated (Holland, 2006; Mills et al., 2022). Diplonemid life cycles turn to be surprisingly complex (Tahyreva et al., 2022). Environmental change is likely a cue for cellular differentiation, as it is in many microbial eukaryotes. Most spectacularly in methylotrophic yeasts a shift in nutrient availability is the cue for metabolic reprogramming that triggers biogenesis of peroxisomes with their own atypical metabolic profile, while redundant ones are turned over en mass through a specific form of autophagy called pexophagy (reviewed in (Manjithaya et al., 2010; Yoshimoto et al., 2010) and discussed in (Hannaert et al., 2003b; Michels et al., 2005; Gualdrón-López et al., 2012a)). Pexophagy occurs in trypanosomatids (Herman et al., 2008; Cull et al., 2014); we contend any hypothesis about the origin of glycosomes should be compatible with the conserved phenomenon of pexophagy. Thus, the wide variety of environments in which extant euglenozoans are found, with inherent variation of nutrient or oxygen availability evident, coupled to complex life cycles, suggests an euglenozoan ancestor would also have lived amid dynamic environmental conditions that could have favoured development of atypical peroxisomes or glycosomes. In response to specific cues, evolution of such atypical peroxisomes might also have been favoured by their ability to be subject to rapid, efficient turnover. Taking note of the availability of numerous euglenozoan genomes and transcriptomes, we have used bioinformatics to attempt to chronologise how the metabolic complexity seen in extant trypanosomatid glycosomes arose.

### Bioinformatic chronology of glycosomal metabolism I: Carbohydrate and energy metabolism

**FIGURE 2:**
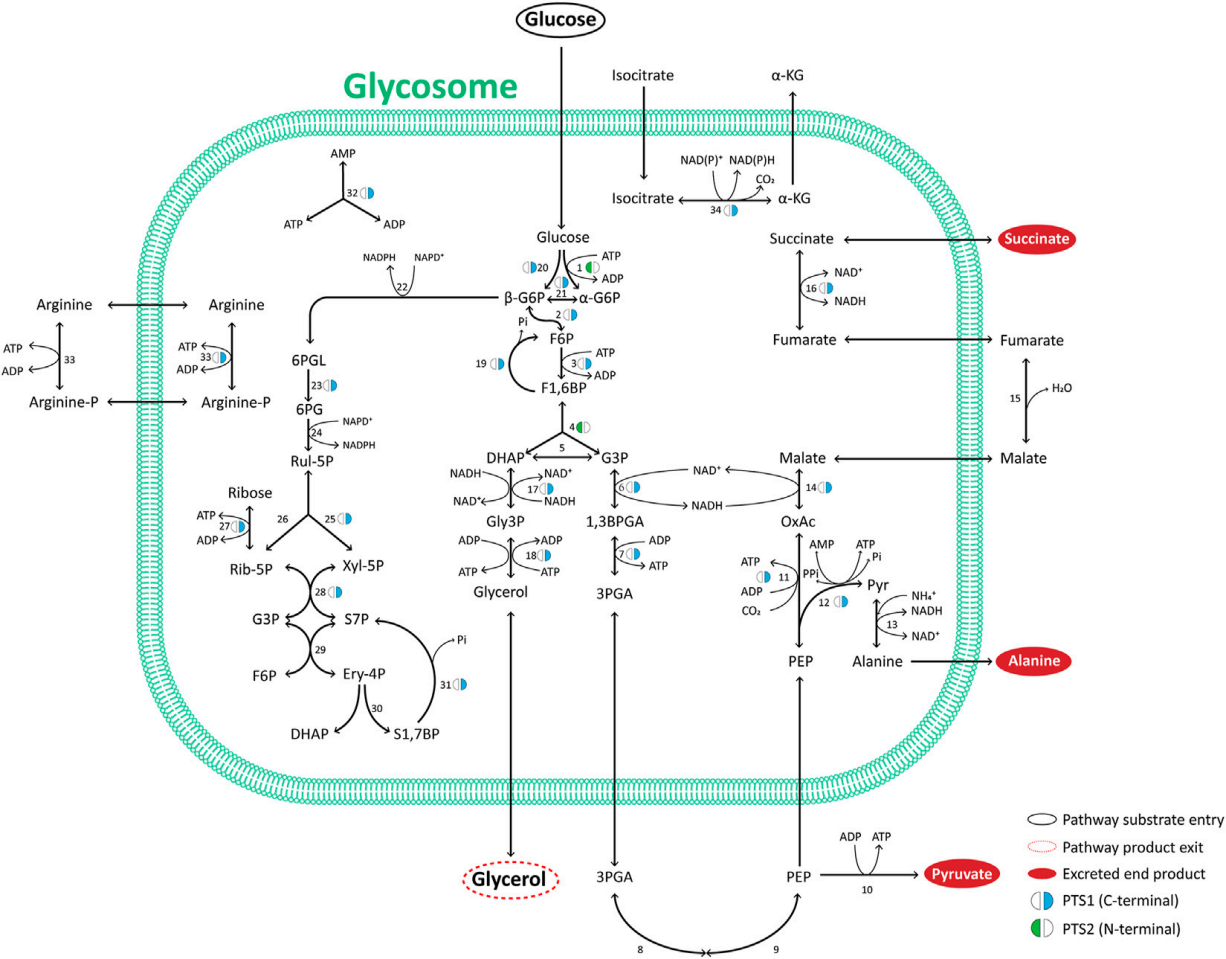
Major pathways of glycosomal carbon metabolism in TriTryps species. Indicated are enzymes of glycolysis, its auxiliary branches, gluconeogenesis, glycerol metabolism and the pentose-phosphate pathway. Note that not all enzymes indicated are present in each of the TriTryps species or expressed in each of their life-cycle stages. Enzymes: **1**. Hexokinase; **2**. Glucose-6-phosphate isomerase; **3**. Phosphofructokinase; **4**. Aldolase; **5**. Triose-phosphate isomerase; **6**. Glyceraldehyde-3-phosphate dehydrogenase; **7**. Phosphoglycerate kinase; **8**. Phosphoglycerate mutase; **9**. Enolase; **10**. Pyruvate kinase; **11**. Phosphoenolpyruvate carboxykinase; **12**. Pyruvate phosphate dikinase; **13**. Alanine dehydrogenase; **14**. Malate dehydrogenase; **15**. Fumarate hydratase; **16**. NADH-dependent fumarate reductase; **17**. Glycerol-3-phosphate dehydrogenase; **18**. Glycerol kinase; **19**. Fructose-1,6-bisphosphatase; **20**. Glucokinase; **21**. d-Hexose-6-Phosphate-1-epimerase; **22**. Glucose-6-phosphate dehydrogenase; **23**. 6-Phosphogluconolactonase; **24**; 6-Phosphogluconate dehydrogenase; **25**. Ribulose-5-phosphate epimerase; **26**. Ribulose-5-phosphate isomerase; **27**. Ribokinase; **28**. Transketolase; **29**. Transaldolase; **30.** Sedoheptulose-1,7-phosphate transaldolase; **31**. Sedoheptulose-1,7-bisphosphatase; **32**. Adenylate kinase; **33**. Arginine kinase; **34**. Isocitrate dehydrogenase. The presence of a PTS1 or 2, the carbon sources used, and the excreted end products are indicated.

The hallmark feature of glycosomes is the presence of most enzymes of the glycolytic pathway in its matrix. In addition, these organelles may contain other enzymes of carbohydrate metabolism, such as those in gluconeogenesis, the PPP as well as in glycerol metabolism. Figure 2 summarizes the glycosomal carbon metabolic network that is representative of most trypanosomatids studied experimentally to date. Most of the enzymes involved contain a consensus PTS. We find this situation is largely conserved in all kinetoplastids and diplonemids analysed (Supplementary Table S1), but not in euglenids or *Naegleria* spp., although the latter possess respectively one or two glycolytic isoenzymes with a predicted PTS. The PTS sequences of kinetoplastid and diplonemid enzymes may differ between orthologues, and in some species no typical PTS can be detected in an orthologue, suggesting that glycosomal targeting is then achieved differently, although an extraglycosomal localisation cannot be excluded. Overall, the data indicate glycolysis from hexokinase (HK) to phosphoglycerate kinase (PGK) must have been compartmentalised in a common ancestor of kinetoplastids and diplonemids and has been retained thereafter.

We note several other interesting evolutionary aspects about glycosomal glycolytic enzymes. First, most kinetoplastids and diplonemids contain both a glycosomal HK with a PTS2 and a glucokinase (GlcK) with a PTS1. However, in some trypanosomatid species and the bodonids a HK without PTS was detected (although in the case of *B. saltans* this could be due to an incomplete sequence (Opperdoes et al., 2016)), while the GlcK has been lost from African trypanosomes (Cáceres et al., 2007). In all species analysed, at least one of the two hexose-phosphorylating enzymes with a PTS is present. HK and GlcK have different anomer preferences: HK for α-glucose, GlcK for the β anomer (Cordeiro et al., 2007). Since enzymes using the product glucose 6-phosphate as substrate, glucose-6-phosphate isomerase in glycolysis and glucose-6-phosphate dehydrogenase in the PPP, have a specificity for the β anomer, a glucose-6-phosphate 1-epimerase is required for interconversion of the anomers. This enzyme with a PTS1 is detected in the databases for all kinetoplastids analysed except *P. confusum*, but not in those of diplonemids and euglenids. Interestingly, it is also found with a PTS1 in *Naegleria* species. Glycosomes of an ancestral kinetoplastid probably acquired the enzyme shortly after divergence from diplonemids, whereas compartmentalisation in peroxisomes of *Naegleria* may have been an independent event. Second, trypanosomatids may contain different isoenzymes for PGK, in glycosomes and the cytosol caused by gene duplications (Rojas-Pirela et al., 2020). Previously we identified a PGK with an N-terminally fused PAS domain associated with *T. cruzi* glycosomes (Rojas-Pirela et al., 2018). PAS domains are involved in signalling, acting as sensors binding small molecules or proteins, but the function of such domain linked to an active PGK is unknown. This PAS-PGK appears to be present in all kinetoplastids, except African trypanosomes that have lost it, and seems to have arisen shortly after the divergence of kinetoplastids and diplonemids. Third, PFK has undergone a peculiar evolution that we consider separately.

Like glycolytic enzymes, a glycosomal gluconeogenic FBPase appears likely in all kinetoplastids and diplonemids as inferred from the presence of a PTS1. The same is true for two enzymes of glycerol metabolism, glycerol kinase and glycerol-3-phosphate dehydrogenase, although the latter was not detected with a PTS in bodonid databases. These enzymes are functionally closely linked with both glycolysis and gluconeogenesis. Furthermore, in the TriTryps, phosphoenolpyruvate produced in glycolysis can be further metabolised in three ways: to pyruvate by either cytosolic pyruvate kinase or glycosomal pyruvate phosphate dikinase (PPDK), or to succinate via the so-called succinic branch comprising glycosomal PEPCK, malate dehydrogenase (MDH) and fumarate reductase (FRD). The fumarate hydratase (FH) of the TriTryps is located in the cytosol, implying a cytosolic loop in the glycosomal succinic branch (Figure 2). Whereas a PTS1 is present in the PPDK in all kinetoplastids and diplonemids analysed, the situation seems more complex for the enzymes of the succinic branch. PEPCK with a PTS1 is present in most representatives of the kinetoplastids, but not diplonemids. MDH with a PTS1 is present only in trypanosomatids, not bodonids and diplonemids, and FRD with a PTS1 is detected in all. It suggests fumarate reduction inside glycosomes is important for kinetoplastids and diplonemids and may already have been rerouted in their common ancestor. But unless current information in the databases is incomplete or alternative targeting signals are present in orthologues, the cytosolic fumarase loop is expanded with one or two neighbouring enzymes in different species. We anticipate the carbon metabolism network would not be substantially affected by rerouting these enzymes because of the existence of pores in membranes of peroxisomes, including glycosomes, considered to allow passage of solutes (inorganic ions and metabolites) with a molecular mass up to approximately 400 Da (Antonenkov and Hiltunen, 2012; Gualdrón-López et al., 2012b; Quiñones et al., 2020; Chornyi et al., 2021; Michels and Gualdrón-López, 2022. The permissiveness of solute passage through peroxisomal membranes is a second key criterion with which any hypothesis about the origin of glycosomes should be compatible. Since the enzymes of the succinic branch use ATP, ADP and NAD(H) as cofactors, which will not pass through the pores, the selective force for their re-routing may be sought in the specific manner by which the different species maintain their intraglycosomal ATP/ADP and redox homeostasis.

The PPP and several of its enzymes have been studied in detail in the TriTryps species (reviewed by Kovářová and Barrett, 2016). It has a dual localisation, with the largest fraction of each enzyme in the cytosol and a smaller part in the glycosomes. Moreover, the size of the glycosomal fraction can differ considerably between the enzymes. Activities and the extent of compartmentalisation of the enzymes vary also with environmental conditions and during the life cycle of the parasites. However, only some of the many PPP enzymes possess a PTS. Transketolase contains a PTS1 in almost all trypanosomatid sequences analysed, but not in those of the bodonids and diplonemids. Regarding 6-phosphogluconolactonase, only the *T. brucei*, *B. saltans* and *D. papillatum* enzymes have a detectable PTS1, while for ribokinase potential PTS1 motifs were detected only in the enzymes from *T. cruzi*, *T. borreli* and *D. japonicum*. Previously, a ribulose-5-phosphate epimerase (RPE) isoenzyme with a PTS1 was detected in *T. cruzi* glycosomes (Gonzalez et al., 2017) and a gene coding for a RPE with a PTS1 found in *D. papillatum* (Škodová-Sveráková et al., 2021). We identified PTS1-containing sequences also in the databases of several other trypanosomatids and *B. saltans*. Together, the data suggest the PPP must have acquired its dual localization early in the evolution of the kinetoplastids/diplonemids, but details of the evolutionary scenario are difficult to trace due to the paucity of consensus glycosomal targeting motifs among PPP enzymes. Major roles of the glycosomal PPP may be to provide NADPH within the organelles for anabolic processes and oxidative defence.

In *T. brucei*, a gene encoding a sedoheptulose-1,7-bisphosphatase (SBPase) with a PTS1 was detected previously (Hannaert et al., 2003a). SBPase is classically an enzyme of the Calvin cycle, not present in trypanosomes, but it has been hypothesised that the enzyme could function in a non-conventional form of the non-oxidative branch of the parasite’s PPP (Hannaert et al., 2003b). It is currently not known if trypanosome SBPase has a dual localisation similar to the PPP enzymes. Our bioinformatics analysis did not identify a SBPase in diplonemids or kinetoplastids, aside from different *Trypanosoma* species, *P. confusum* and *B. saltans*. When present, it always possesses a PTS1. Phototrophic *E. gracilis* contains a SBPase for the Calvin cycle in its plastid; its two 75% identical subunits are encoded as biprotein precursor that must have originated from a gene duplication. The protein is very different from that found in kinetoplastids (Teich et al., 2007). No orthologue of the kinetoplastid SBPase was detected in *N. gruberi* (Opperdoes et al., 2011). These results suggest kinetoplastids gained SBPase through lateral gene transfer.

Isocitrate dehydrogenase (IDH) with a PTS1 has been detected in *T. brucei* and *T. cruzi*. The enzyme could possibly function as an extra mechanism for providing intraglycosomal NAD(P)H. Euglenozoans, like other eukaryotes, possess multiple IDH isoenzymes but in this protist group the gene family has gone through a puzzling evolution. As with euglenozoan PFK, we consider IDH evolution in a subsequent section.

Nucleotide-sugar biosynthesis has been identified as a glycosomal process in the TriTryps. In *T. brucei*, glucose is converted into five essential nucleotide sugars, required for the synthesis of glycoconjugates: UDP-Glc, UDP-Gal, UDP-GlcNAc, GDP-man and GDP-Fuc by 13 enzymes, most of them present in glycosomes, five of them having a predicted PTS (Sampaio-Guther et al., 2021). The process occurs also within glycosomes of *T. cruzi* and *Leishmania* spp., as inferred from the presence of PTS motifs in several of its enzymes (Sampaio-Guther et al., 2021) (Supplementary Figure S1 and Supplementary Table S1). In these latter parasites, galactose can also serve as substrate for the synthesis of nucleotide sugars, because of the presence in their glycosomes of both a galactokinase (phosphorylating the sugar at its C1 position) and an UDP-pyrophosphatase; these enzymes are absent from *T. brucei* (Lobo-Rojas et al., 2016; Michels et al., 2021). Orthologues of several enzymes of nucleotide-sugar biosynthetic enzymes with a PTS can be detected in each trypanosomatid, bodonid and diplonemid analysed, but not in euglenids or *Naegleria* spp. The process was thus probably compartmentalised already in the common ancestor, simultaneously with or shortly after the routing of glycolytic/gluconeogenic enzymes to the organelles. No galactokinase orthologue was found in diplonemids, suggesting that the possibility to use this sugar was only acquired by kinetoplastids. Yet, absence of this kinase from African trypanosomes, plus the fact it was not found in the databases of some other kinetoplastids–*P. confusum* and *B. ayalai*–suggests either acquisition or loss on multiple occasions.

### Euglenozoan PFKs–surprisingly complex evolutionary histories

The evolution of PFK in Euglenozoa is intriguing. Homology exists between the majority of PFKs in nature, but eight different clades can be distinguished, some comprising mainly enzymes that use ATP as phospho donor, while in other groups most PFKs act with PPi (Mertens et al., 1998; Bapteste et al., 2003). Previously, the enzyme in *T. brucei* and the other TriTryps was phylogenetically shown to belong to a group consisting predominantly of PPi-PFKs, although it is catalytically strictly dependent on ATP. During its evolution, the enzyme must have undergone mutations that changed its phospho-donor specificity (Michels et al., 1997; McNae et al., 2009). Such changes have also been reported for other lineages of microorganisms (Bapteste et al., 2003). This can occur easily, as was experimentally demonstrated; substitution of only two specific active-site residues of the *Entamoeba histolytica* PPi-PFK were sufficient to change a 10^6^-fold preference for PPi over ATP (calculated as the ratio of *k*_cat_/*K*_*m*_) into a 10^2^-fold preference for ATP (Chi and Kemp, 2000). An essential difference between ATP and PPi catalysed PFK reactions is that the former is virtually irreversible under physiological conditions, whereas the latter readily carries out both forward and reverse reactions, and thus can also replace the FBPase in the gluconeogenic pathway. In glycosomes, where under certain conditions a low ATP/ADP ratio can occur independently of the ratio in the cytosol, it may be possible to also reverse the ATP-PFK reaction (Fernandes et al., 2019; Michels et al., 2021).

**FIGURE 3:**
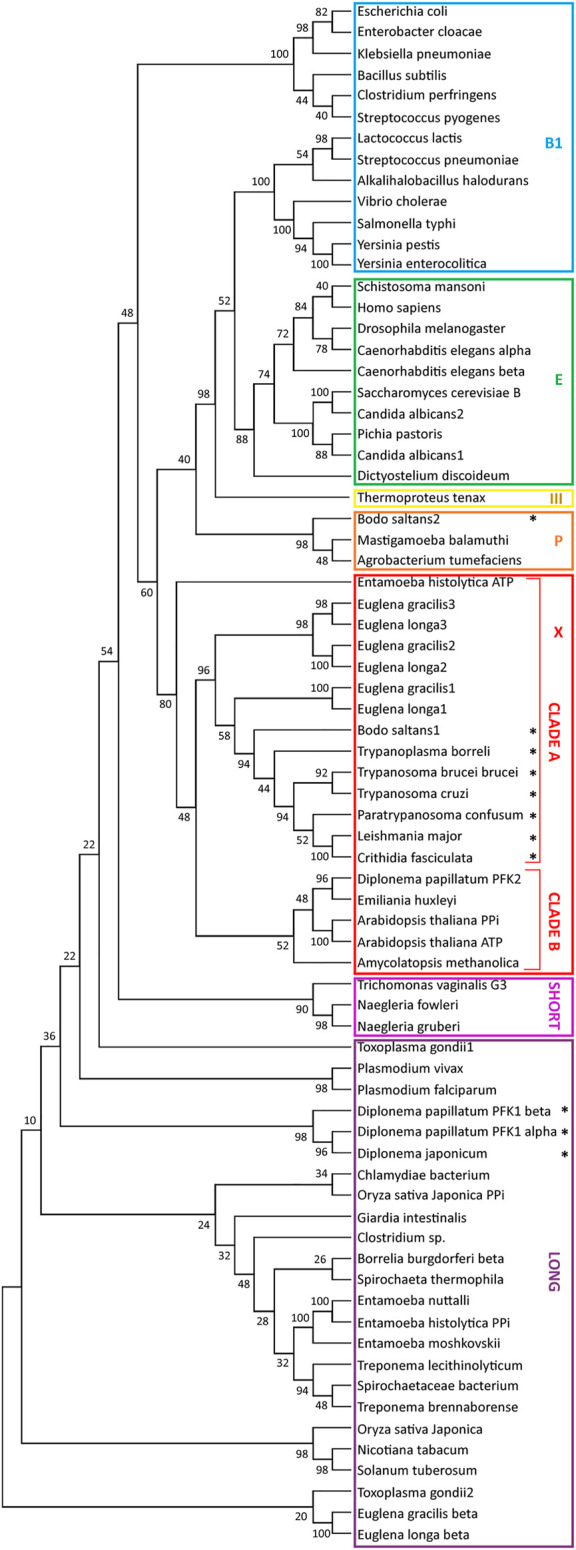
Relationships between phosphofructokinase (PFK) sequences. The evolutionary history of PFK sequences was inferred by from their amino-acid sequences by using the Maximum Likelihood method and JTT matrix-based model (Jones et al., 1992). For each enzyme analysed, a bootstrap consensus tree inferred from 500 replicates (Felsenstein, 1985) was taken to represent the evolutionary history (Felsenstein, 1985). Branches corresponding to partitions reproduced in less than 50% bootstrap replicates were collapsed. The percentage of replicate trees in which the associated taxa clustered together in the bootstrap test (50 replicates) are shown next to the branches of the trees (Felsenstein, 1985). Initial tree(s) for the heuristic search were obtained automatically by applying the Maximum Parsimony method. All positions with less than 95% site coverage were eliminated, *i.e.*, fewer than 5% alignment gaps, missing data, and ambiguous bases were allowed at any position (partial deletion option). Evolutionary analyses were conducted in MEGA X (Kumar et al., 2018). The analysis involved 75 amino-acid sequences, with a total of 242 positions in the final dataset. The stars indicate sequences with a PTS1. Sequences used for the construction of the tree were retrieved from the following databases: NCBI, TriTrypDB and AmoebaDB and uploaded in FASTA format. The sequences were aligned with Clustal and the alignment was manually refined with the program MEGA X. The nomenclature of the clades was taken from Bapteste et al., 2003.

Each TriTryp PFK contains a PTS1 and is found in glycosomes. Our bioinformatic analysis revealed homologous PFKs with a PTS1 can be detected in all other kinetoplastids analysed, except in *Leptomonas* species where the enzyme does not contain a consensus PTS motif (Supplementary Table S1). Furthermore, we detected in the transcriptomes of the euglenids *E. gracilis* and *E. longa* three sequences encoding related (appr. 50 – 65% identity) PFKs, each without PTS. They share 47–48% identity with different kinetoplastid PFKs and phylogenetically clustered with them in a clade previously called X (Bapteste et al., 2003) containing mostly PPi-PFKs as well as some ATP-dependent enzymes (Figure 3). These *Euglena* PFKs are predicted to have ATP-dependent activity as we conclude from the conservation of residues known to be involved in the binding of this phospho donor in trypanosomes (McNae et al., 2009). In addition, a very different PFK was found in the euglenid databases, annotated as β-PFK. Plants and algae including *E. gracilis* contain three PFKs: ATP-PFK and PPi-PFK in the cytosol, and another ATP-PFK in the chloroplast (Mertens, 1991). The PPi-PFK is usually a heterotetramer consisting of large homologous catalytic (β) and regulatory (α) subunits, which are often about 35% identical. A homologous heteromeric PPi-PFK is present in some protists and prokaryotes. Indeed, this *Euglena* β-PFK is phylogenetically found with these PPi-PFKs in a clade called ‘Long’ (Mertens et al., 1998; Bapteste et al., 2003) (Figure 3).

In contrast, we did not detect an orthologue of the kinetoplastid/euglenid PFK for diplonemids in the databases, in agreement with previous reports by Morales et al. (2016) and Škodová-Sveráková et al., 2021. The *D. papillatum* genome database contains three PFKs, one called DpPFK2, that shows most identity (about 36%) with the PFK of the algal haptophyte *Emiliana huxleyi* and belongs to ‘clade B’ alongside principally algal and plant PFKs. Identity with kinetoplastid PFKs is less than 30%, and there is no consensus PTS motif (Morales et al., 2016). We assume it was acquired by horizontal gene transfer (Škodová-Sveráková et al., 2021). The two other *D. papillatum* sequences, one originally identified (Morales et al., 2016) and called DpPFK1 (classically also denoted as PFP for PPi-fructose 6-phosphate 1-phosphotransferase) and a related one, identified later (Škodová-Sveráková et al., 2021) are similar to each other (35% identity) and each contains a PTS1 (-PKL). They show most, but still relatively low identity, with α and β subunits of plant/algal/prokaryote PPi-PFKs, including euglenid β-PPi-PFK (30 and 27%), with which they display only a distant relationship by phylogeny. Therefore, it seems unlikely that *Diplonema* inherited a heteromeric PPi-PFK from the common Euglenozoa ancestor. More plausible is a bacterial origin, as also proposed previously (Škodová-Sveráková et al., 2021), for example by acquisition of a DNA segment containing (part of) an operon with the genes of the two subunits, followed by creation of topogenic signals to route the gene products into glycosomes. Inspection of the sequences using information from the crystal structure of *Borrelia burgdorferi* PPi-PFK (Moore et al., 2002) indicated one of these *D. papillatum* proteins contains all essential residues for PPi-dependent PFK activity, thus probably is the β-subunit, whereas the other lacks many residues required for activity and is thus a likely α-subunit candidate.

A comparable although more complicated situation is found in the distantly related diplonemid *H. phaeocysticola* (Tashyreva et al., 2022) where we identified six putative PFK sequences in its transcriptome database (not shown). Two belong to the ‘Long’ clade, with one possessing a PTS1 (-AHL) and being mostly similar to the *D. papillatum* β-PFK1 and less to *E. gracilis* β-PFK (51 and 31% identity, respectively). The other sequence is very different, sharing 34% identity with the putative β-HpPFK and less than 20% with both α- and β-DpPFK1 and β-EgPFK. The other four Hp sequences are smaller: two have a PTS1 (-PKL and -SRM), contrary to the corresponding DpPFK2. Three of the sequences are 57–61% identical to each other and all are more similar to DpPFK2 and *E. huxley* PFK than to kinetoplastid PFKs. Together, the data about *H. phaeocysticola* PFK corroborate the evolutionary scenario depicted for *Diplonema* spp.

In summary, kinetoplastids undoubtedly inherited the cytosolic ‘clade X’ PFK from a common ancestor with *Euglena* and compartmentalised it, together with other glycolytic enzymes, into their peroxisomes that so became glycosomes. In contrast, they lost the ‘clade Long’ PPi-PFK that was probably present in the ancestor. PFK ‘clade X’ of the common Euglenozoa ancestor was almost certainly ATP dependent, indicating phospho-donor specificity had already changed from PPi to ATP at an earlier evolutionary stage. Diplonemids seem to have lost the PFKs of both clades but acquired others by horizontal gene transfer, retaining the PPi-dependent activity of the enzyme that became sequestered in the glycosomes.

Intriguing questions raised by the proposed evolutionary scenario are why an ancestral diplonemid acquired different PFKs by horizontal gene transfer while having lost its ancestral ones, and why are both new PFKs retained in different progeny lineages. What has been the evolutionary driver to maintain a PPi-PFK together with a (probable) ATP-PFK and FBPase? Perhaps adaptation to possible anoxic conditions played a role with a somewhat higher glycolytic ATP yield with a PPi-PFK compared to ATP-PFK (Mertens 1993; Saavedra et al., 2019). Alternatively, there is conceivably a large requirement for gluconeogenesis when these diplomemids rely on amino acids as nutrients and need to synthesize purines, pyrimidines and ribose-phosphates for their massive kinetoplast DNA networks (Lukeš et al., 2018). Answers to the questions posed will require more information about the kinetic and activity-regulated properties of the ATP-PFK, FBPase and PPi-PFK, and their expression levels under different conditions.

Intriguingly, the eubodonid *B. saltans* contains, in addition to a PTS1 (-SKL) containing PFK related to the glycosomal PFK of all other kinetoplastids, a very different PFK (25% identity). It also has a PTS1 (-SRL) (Opperdoes et al., 2016). No similar sequence was detected in any other kinetoplastid, including the parabodonid *T. borreli* and the early-branching intracellular kinetoplastid *Perkinsela* sp. It was presumably acquired by horizontal gene transfer. However, it is doubtful this protein has PFK activity because several typical substrate (ATP/PPi and fructose 6-phosphate) binding residues have been substituted. The possible function of this PTS1-containing protein is unknown.

### Euglenozoan isocitrate dehydrogenase families–Diverse evolutionary trajectories

**FIGURE 4:**
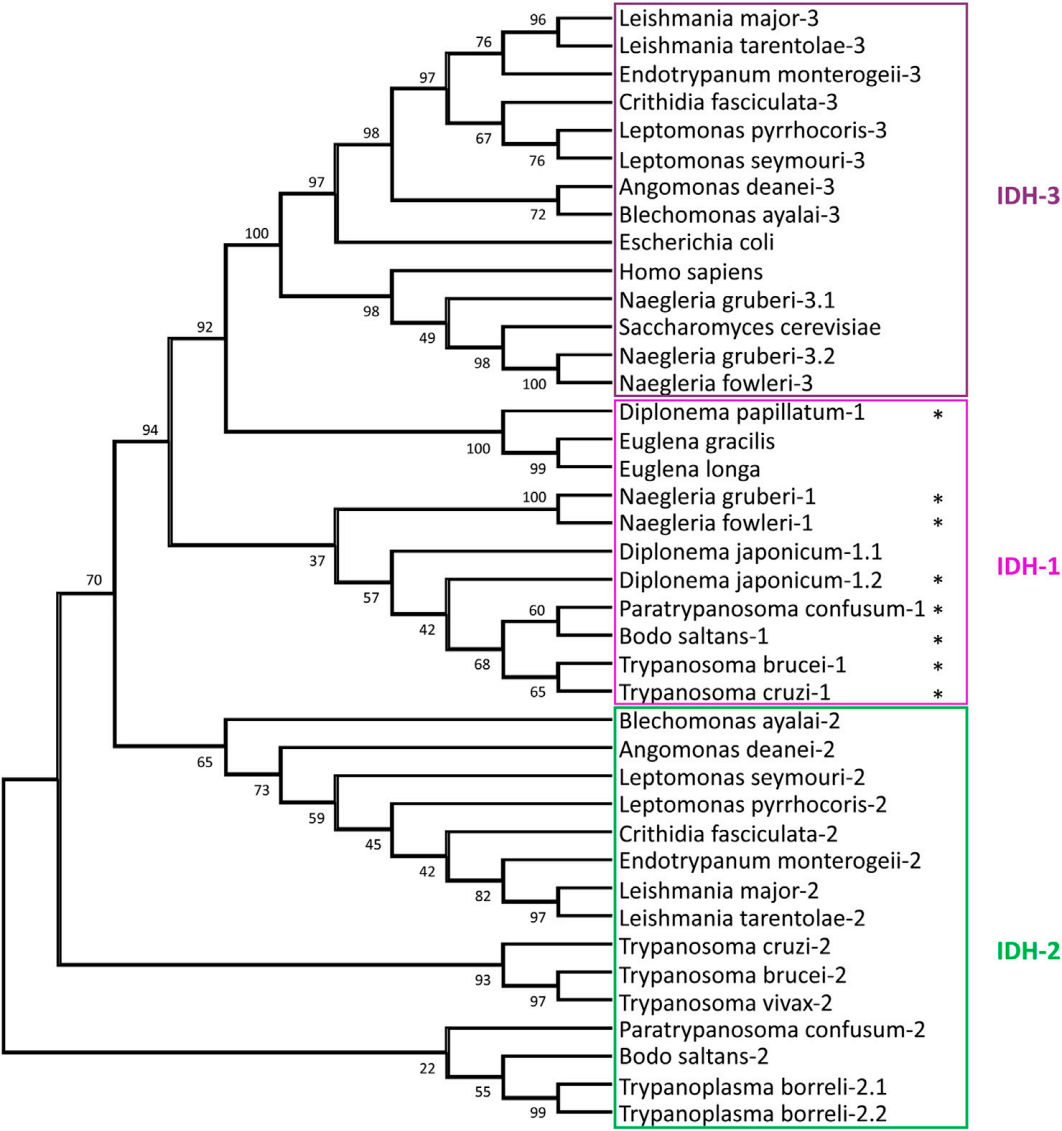
Relationships between isocitrate dehydrogenase (IDH) sequences. The evolutionary history of IDH sequences was inferred by methods described in the legend of Figure 3. The analysis involved 40 amino-acid sequences, with a total of 319 positions in the final dataset. The stars indicate sequences with a PTS1. The following IDH sequences were retrieved from the NCBI: *H. sapiens*, *S. cerevisiae*, *E. coli*, *T. borreli*, *D. papillatum*, *D. japonicum*, *E. longa*, and *E. gracilis*, whereas for *Naegleria* spp. the sequences were retrieved from AmoebaDB. The other euglenozoan sequences were taken from TriTrypDB.

Three different types of IDHs are generally distinguished in eukaryotes: an NADP^+^-dependent IDH1 in the cytosol and/or peroxisomes and NADP^+^- and NAD^+^-dependent IDH2 and IDH3, respectively, in mitochondria. IDH1 and IDH2 show considerable similarity, whereas IDH3, which functions in the tricarboxylic acid (TCA) cycle, is very different and most related to prokaryotic NADP^+^/NAD^+^ IDHs. The *T. brucei* genome contains two genes, coding for IDH1 and IDH2, enzymes which are 62% identical. Its IDH1 has a PTS1 (-SKV) and the IDH2 a predicted N-terminal mitochondrial-targeting signal (MTS). Only the IDH1 has been experimentally characterised (Wang et al., 2017) and, surprisingly, showed activity with both NADP^+^ and NAD^+^, even higher with the latter cofactor. The orthologues in *T. cruzi*, which were both shown to be strictly NADP^+^-dependent, have also a PTS1 and MTS, respectively. Whereas the IDH2 was indeed located in the mitochondrion, essentially all IDH1 was, in epimastigotes, in the cytosol (Leroux et al., 2011). No evidence was found for the typical TCA cycle-related NAD^+^-dependent IDH3 in the *Trypanosoma* species. In *L. mexicana* two IDH isoenzymes are also found: one, with an MTS and high NADP^+^ specificity is most related to IDH2 of trypanosomes, whereas the other exclusively uses NAD^+^, and is very different (Giordana and Nowicki, 2020). This isoform contains no strong targeting motif, and appears most related to the TCA-cycle IDH3 enzyme of bacteria and other eukaryotes. Both *Leishmania* IDHs exhibit dual localisation in the mitochondrion and cytosol. Orthologues of both *Leishmania* IDHs were reported for *Leptomonas* and *Crithidia*. Our bioinformatic analysis did also not identify IDH1 orthologues in this *Leishmania*/*Leptomonas*/*Crithidia* group, nor in *Endotrypanum*, *Angomonas* or *Blechomonas*. However, IDH1 with a PTS1 was, additionally to *Trypanosoma* species, also detected in the kinetoplastids *P. confusum* and *B. saltans,* in diplonemids and in *Naegleria*, but not in *Euglena* (Supplementary Table S2). Figure 4 shows a phylogenetic analysis of all IDHs detected in the genome databases of all these Discoba protists. Data about *Euglena* and *Diplonema* species are probably still incomplete. The clustering of the IDH1s of *Naegleria*, *Diplonema* and kinetoplastids reveals glycosomes retained this isoform with its characteristic partial organellar localisation when they evolved from peroxisomes, but a common ancestor of the *Leishmania*/*Leptomonas*/*Crithidia/Angomonas/Blechomonas* group must have lost it. In contrast, these kinetoplastids possess a mitochondrial NAD^+^-IDH3. Apparent absence of an IDH3 in the other kinetoplastids and diplonemids, as well as the large difference (about 30% identity) with the *Naegleria* IDH3 indicates IDH3 was obtained by lateral gene transfer from a bacterium. Intriguingly, two very different *N. gruberi* IDH3s, both with an MTS, were detected by in genome and transcriptome databases. We were also surprised to observe two very similar IDH1s (83% identity) but no IDH2 in *D. japonicum*. One sequence contains a PTS1, whereas the other has a potential MTS. We suggest this diplonemid has lost an IDH2 gene but compensated by duplication of the IDH1 gene with adaptation of the required targeting motifs of the encoded proteins. In contrast, in *T. borreli* genes encoding two very similar IDH2s (81% identity) were detected, both with an MTS, but no IDH1 was found.

The finding that, in several kinetoplastids such as trypanosomes, the mitochondrion lacks the NAD-dependent IDH3 in several kinetoplastids, but has a NADP-dependent IDH2, suggests the absence of a fully functional TCA cycle in these organisms, with the other cycle enzymes functioning in metabolising products of fatty acid and amino acid oxidation. IDH2 serves to maintain the mitochondrial NADP^+^/NADPH ratio (Opperdoes et al., 2016).

The function of an IDH within glycosomes remains to be established. It may contribute to the maintenance of the NAD(P)^+^/NAD(P)H balance, with the substrate and product of the reaction, isocitrate and α-ketoglutarate, exchanging across the membrane (Figure 2). The level of expression of this redox shuttle may vary during life-cycle stages and/or environmental conditions, while in some kinetoplastids presence of a glycosomal IDH may have become redundant and lost. Whether there is a functional link between this loss and the acquisition of a mitochondrial IDH3 by lateral gene transfer is not clear. A shuttle involving isocitrate and α-ketoglutarate has also been identified for peroxisomes of yeast and proposed for those of mammalian cells (reviewed by Chornyi et al., 2021).

Finally, an α-ketoglutarate decarboxylase (α-KDE1), the E1 subunit of the TCA-cycle α-ketoglutarate complex, was located in the mitochondrion and glycosomes of *T. brucei* (Sykes et al., 2015). The E2 and E3 subunits are not glycosomal. Here, glycosomal targeting is via an unusual PTS2 motif partially overlapping with the N-terminal MTS (MMRRLSPVNGSV-). Whether this glycosomal α-KDE1 acts on the IDH1 product or if it has an unknown moonlighting function awaits discovery. In addition, variations in the unusual PTS in the α-KDE1 homologue of other euglenids preclude making predictions of conservation of the glycosomal localisation.

### Bioinformatic chronology of glycosomal metabolism II: ATP/ADP/AMP homeostasis

Two other enzymes related to energy metabolism are found in glycosomes: adenylate kinase (ADK) and arginine kinase (AK). ADKs contribute to homeostasis of the adenine nucleotide pool by reversible phosphoryl transfer resulting in the interconversion of two ADP molecules and ATP plus AMP (Figure 2). Trypanosomes contain multiple ADKs, including one in glycosomes (Ginger et al., 2005). In all kinetoplastids analysed an ADK with a PTS1 was detected (Supplementary Table S1). Morales et al. (2016) concluded from phylogenetic analysis that kinetoplastids had acquired the glycosomal isoform by lateral gene transfer from a bacterium. However, we detected an ADK with a PTS1 in *Diplonema* spp.*,* but not in *Euglena* and *Naegleria*, suggesting that the acquisition occurred earlier.

AKs catalyse the reversible phosphorylation of arginine by ATP. Phosphoarginine provides an energy reserve in many invertebrates and in some unicellular eukaryotes. *T. brucei* contains three highly similar AK isoforms located in the flagellum, cytosol and glycosomes, the latter has a PTS1 (Voncken et al., 2013). A PTS1 containing AK was also found in *T. vivax*. Only glycosomes of African trypanosomes acquired an AK, possibly creating a relay system between cytosol and glycosomes allowing the maintenance of the intraglycosomal ATP/ADP balance when required without the need for transfer of adenine nucleotides across the organellar membrane. *T. cruzi* has two AK isoforms, each without a PTS and not detected in the organelles (Miranda et al., 2009). Genes encoding AK orthologues were found only in genomes of a few other kinetoplastids (*Blechomonas ayalai* and *Bodo saltans,* in each case without PTS). Sequences annotated as arginine or creatine kinases were also detected in diplonemid databases, with an enzyme in *D. ambulator* (27–29% identity with *Trypanosoma* AKs) containing a PTS1.

It has been proposed that absence of AK in several species is linked to the presence of the arginine-consuming enzyme arginase (ARG); simultaneous activity of AK and ARG within a cell should be incompatible (Canepa et al., 2011). Indeed, *Trypanosoma* and other trypanosomatids such as *Phytomonas* that possess an AK lack a functional ARG, whereas the opposite situation is found in Leishmaniinae (*Leishmania*, *Crithidia*, *Endotrypanum*, *Leptomonas*) and *Angomonas*. However, *B. saltans* contains, in addition to a predicted AK gene, a potential ARG with a PTS1 (Opperdoes et al., 2016). Confirmation of whether this protein has arginase activity is required. Preventing incompatibility of the ARG and AK by differential regulation at gene expression and/or activity level can also not be excluded. The *B. saltans* ARG is not orthologous to the Leishmaniinae ARG (Opperdoes and Michels 2008; Opperdoes et al., 2016) that is glycosomal with a PTS1 in all species analysed (Roberts et al., 2004). Phylogenetic analysis suggested that the Leishmaniinae gene was acquired by lateral transfer from fungi. Arginases convert arginine to ornithine, a precursor for polyamine biosynthesis. Kinetoplastids without this enzyme, like *Trypanosoma* species, rely on the uptake of ornithine (Marchese et al., 2018). The evolution of AK and ARG in Euglenozoa probably involved multiple acquisition and losses of genes. Likelihood of independent acquisition of glycosomal targeting requires further study.

### Bioinformatic chronology of glycosomal metabolism III: Lipid metabolism

**FIGURE 5:**
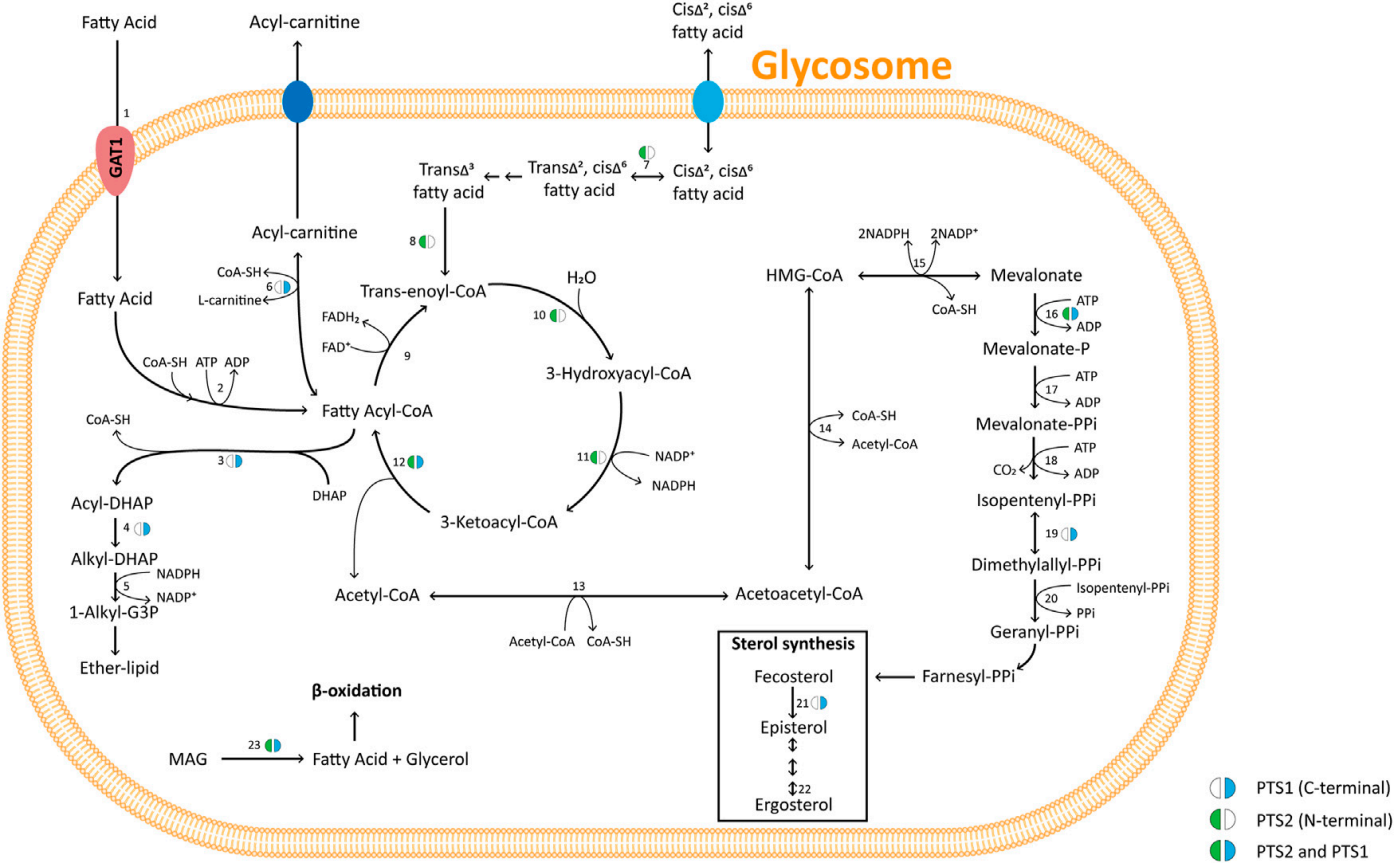
Pathways of glycosomal lipid metabolism in TriTryps species. Pathways of ether-lipid synthesis (enzymes 3 – 5), fatty-acid β-oxidation (9 – 12), and sterol synthesis (16 – 23) are represented. Note that not all enzymes indicated are present in each of the TriTryps species or expressed in each of their life-cycle stages, and for simplicity not all reactions are represented. Enzymes/transporter: **1.** Glycosomal ABC transporter (GAT1); **2.** Fatty-acyl CoA synthetase; **3**. DHAP acyltransferase; **4**. Alkyl-dihydroxyacetone phosphate synthase; **5**: 1-Alkyl G3P:NADP^+^ oxidoreductase; **6**. Carnitine O-palmitoyltransferase; **7.** 3,2-Trans-enoyl-CoA isomerase; **8.** Enoyl-CoA isomerase; **9**. Acyl-CoA dehydrogenase; **10, 11, 12.** Enoyl-CoA hydratase/3-hydroxyacyl-CoA dehydrogenase/3-ketoacyl-CoA thiolase (trifunctional enzyme); **13**. Acetyl-CoA acetyltransferase **14**. 3-Hydroxy-3-methylglutaryl CoA synthase; **15**. 3-Hydroxy-3-methylglutaryl-CoA reductase; **16**. Mevalonate kinase; **17**. Phosphomevalonate kinase **18**. Mevalonate diphosphate decarboxylase **19.** Isopentenyl-diphosphate δ-isomerase; **20**. Farnesyl diphosphate synthase **21**. C-8 sterol isomerase; **22**. Sterol C-24 reductase; **23.** Monoacylglyceride lipase. **MAG**: monoacylglycerol. Enzymes **13, 14, 15, 17, 18** and **20** have been detected experimentally with a PTS motif in mammalian peroxisomes (references cited in Acosta et al., 2019).

Peroxisomes play a major role in the lipid metabolism of many eukaryotes. This is also true for trypanosomatid glycosomes as previously shown in several functional and proteomic studies (reviewed by Allmann and Bringaud, 2017) and other euglenozoans as demonstrated in this work (Figure 5 and Supplementary Table S2). Different enzymes of sterol metabolism (C-8 sterol isomerase, isopentenyl-diphosphate δ-isomerase, mevalonate kinase) are detected throughout the kinetoplastid lineage with either a type 1 or 2 PTS, while some of these enzymes containing a PTS are found in databases of *Diplonema* spp., *Euglena* spp. and *Naegleria* spp.

Enzymes involved in the formation of fatty acids from phospholipids and their oxidation have also been detected in *Trypanosoma* and *Leishmania* glycosomes. Several are found with a PTS in many euglenozoans and in *Naegleria* spp. monoglyceride lipase, acyl-CoA binding protein, carnitine O-palmitoyltransferase, and each of the β-oxidation enzymes. This indicates all were peroxisomal ancestrally in the kinetoplastid/diplonemid progenitor before glycolysis became sequestered in the organelles.

Similar to mammals, β-oxidation in kinetoplastids occurs in both peroxisomes/glycosomes and mitochondria. In plants and yeast, β-oxidation occurs only in peroxisomes. There is a major difference between mitochondrial and peroxisomal β-oxidation: the first oxidative step of the mitochondrial pathway is catalysed by an acyl-CoA dehydrogenase (ACAD) in which electrons are transferred to the respiratory chain, but in peroxisomes this step typically involves an acyl-CoA oxidase (ACOX) that reduces O_2_ to H_2_O_2_. However, often organisms contain different isoenzyme families for both ACADs and ACOXs, and ACADs containing a PTS are functional within peroxisomes in mammals and fungi (Camões et al., 2015). ACADs and ACOXs are related, FAD-containing enzymes (Kim and Miura, 2004). In the *N. gruberi* genome, a putative ACAD with a PTS1 was detected, but no ACOX gene (Opperdoes et al., 2011) and recent analysis of *D. papillatum* revealed four very different transcripts encoding putative ACADs (including one with a PTS1) plus three strikingly different mRNAs for predicted ACOXs (also including one with a PTS1) (Škodová-Sveráková et al., 2021). Candidate ACADs and ACOXs with PTS1 motifs were detected in other diplonemids, euglenids and *Naegleria*, but not in any kinetoplastid (Supplementary Table S2). According to TriTrypDB, kinetoplastids contain different predicted ACADs and ACOXs, but a PTS was only detected in candidate ACOXs of *B. saltans* (Opperdoes et al., 2016) and *P. confusum* with no apparent homologues in the other species. It should be noted that different online informatics resources provided different outcomes for ACAD vs*.* ACOX prediction. Thus, future use of more bespoke software and/or experimental approaches will be required to reach definite conclusions.

While, to our knowledge, no activity of the first step of glycosomal β-oxidation in trypanosomes has been reported, activities have been demonstrated for the other three reactions (Wiemer et al., 1996; Mazet et al., 2011). The second and third enzymes, enoyl-CoA hydratase and 3-hydroxyacyl-CoA dehydrogenase, together with enoyl-CoA isomerase, are present as a trifunctional enzyme, while the last enzyme, 3-ketoacyl-CoA thiolase, is separate.

Thiolases exist in different isoforms with different substrate specificity and metabolic functions. The SCP2-isoform was detected in each TriTryps parasite and was the only isoform detected in *T. brucei*; *T. cruzi* and *Leishmania* spp. contain one and two other isoforms, respectively (Mazet et al., 2011). Trypanosomatid SCP2 isoform contains a candidate PTS1 and at its N-terminus both a predicted MTS and PTS2. The *T. brucei* protein, when expressed as recombinant protein, was shown to be active in both directions, and would thus be able to participate in β-oxidation when required. However, in procyclic trypanosomes it was located in the mitochondrion and shown to be involved in lipid biosynthesis (Harijan et al., 2016). Although glycosomal β-oxidation is potentially possible, it is not yet experimentally demonstrated. The predicted glycosomal targeting situation for the trifunctional enzyme and SCP2 thiolase in Euglenozoa is very similar, with a potential PTS2 in most kinetoplastids, and a PTS1 in diplonemids, euglenids and *N. gruberi*. Moreover, some thiolases, like that of *T. cruzi* and *C. fasciculata* also possess a candidate PTS1.

Enzymes of ether-lipid biosynthesis (dihydroxyacetone-phosphate acyltransferase, alkyl-dihydroxyacetone-phosphate synthase, fatty acyl-CoA reductase) are well-known peroxisomal markers (Opperdoes, 1984) and contain a PTS1 or PTS2 in all Euglenozoa analysed, as well as in *Naegleria*.

Finally, the presence of a novel phospholipase dually localised in the cytosol and glycosomes of *T. brucei* was recently reported (Monic et al., 2022). The enzyme has a non-canonical PTS1 (-SKS) required for glycosomal import, although this motif showed very poor efficiency in previous comparative glycosomal-targeting experiments (Sommer et al., 1992). Moreover, the novel lipase appeared to be excreted by bloodstream-form (BSF) trypanosomes. The possible function of the lipase within glycosomes is unknown. Orthologues with a potential PTS1 were only detected in databases of *T. cruzi* (-SKA) and *D. japonicum* (-SNM).

### Bioinformatic chronology of glycosomal metabolism IV: Antioxidant metabolism

Glycosomal antoxidant metabolism is depicted in Supplementary Figure S2. Strikingly, the hallmark peroxisomal enzyme catalase, responsible for the dismutation of the reactive H_2_O_2_, could never be detected in glycosomes or elsewhere in the cells of TriTryps parasites. It was thought this was due to a reduced peroxide metabolism. Activity of ACOXs and other H_2_O_2_-producing enzymes such as d-amino acid oxidase and 2-hydroxy acid oxidase could not be detected in *T. brucei*, nor have these enzymes been detected in any of the more recent glycosomal proteome analyses. However, the latter two oxidases and catalase have been reported for other trypanosomatids like *C. fasciculata*, but not in association with the organelles (Opperdoes et al., 1977; Gutteridge et al., 1982; Opperdoes, 1987). We detected in TriTrypDB an *A. deanei* oxidase with a FAD-binding domain and PTS1, annotated as having a predicted 2-hydroxy acid oxidase function (Supplementary Table S2). It has homologues with a PTS in most kinetoplastids but was absent from *Trypanosoma* species. A possible d-amino acid oxidase with PTS1 was detected in the database of *B. saltans*, but no obvious homologues of it was detected in other Euglenozoa. These data, together with the predicted presence of ACOXs mentioned in the preceding section, suggest H_2_O_2_ metabolism in glycosomes may be more prominent than appreciated so far.

Catalase, not detected in the TriTryps parasites, appears present in diverse euglenozoans. Many years previously, it was detected in glycosomes of *T. borreli* (Opperdoes et al., 1988). Recently, however, detailed genomic analysis provided evidence for independent catalase gene acquisitions by several Euglenozoa through horizontal transfers to execute specific molecular tasks (Škodová-Sveráková et al., 2020). Genes encoding a catalase with a PTS motif (-SQA) were only reported for two diplonemids–*D. papillatum* and *D. japonicum*–but not in other diplonemids or kinetoplastids.

In the absence of catalase, detoxification of glycosomal H_2_O_2_ may be performed by the trypanothione-based peroxide detoxification system. Although this system is primarily present in the cytosol of trypanosomatids, small amounts of some of its proteins have been found associated with glycosomes (Irigoín et al., 2008; Güther et al., 2014). Moreover, several of these proteins contain a PTS (Opperdoes and Szikora, 2006), conserved in all kinetoplastids and diplonemids. Other enzymes of antioxidant metabolism detected in *Trypanosoma* glycosomes are a PTS1 containing l-galactonolactone oxidase involved in ascorbate synthesis and two iron-containing superoxide dismutase (Fe-SOD) isoenzymes, one of them having a PTS1 (Wilkinson et al., 2005; Dufernez et al., 2006; Wilkinson et al., 2006). Among the Euglenozoa a PTS-containing l-galactonolactone oxidase was only detected in *B. ayalai*, whereas Fe-SODs with a PTS1 are present throughout the phylum.

### Bioinformatic chronology of glycosomal metabolism V: Pyrimidine synthesis, purine salvage

Trypanosomatids perform *de novo* pyrimidine synthesis (Supplementary Figure S3). Most of the six enzymes involved occur in the cytosol. However, the last step of UMP biosynthesis involves two enzymatic activities, orotidine-5′-monophosphate decarboxylase and orotate phosphoribosyltransferase, that in trypanosomatids are carried out by a glycosomal bifunctional enzyme (OMPDC/OPRT) with a PTS1 encoded by a single, fused gene (Gao et al., 1999). Interestingly, a bifunctional enzyme is also present in many other eukaryotes including *Euglena* and *Naegleria*, but with enzymatic domains fused in the reverse order (OPRT/OMPDC) (Makiuchi et al., 2007). Orthologues of the bifunctional enzyme with a PTS1 were detected in all kinetoplastids analysed in this study, but not in diplonemids, euglenids or *Naegleria* spp. (Supplementary Table S2). Butenko et al. (2020) detected separate OMPDC and OPRT transcripts in diplonemids and euglenids, as well as a transcript encoding the fused protein in some euglenids and the diplonemid *Hemistasia phaeocysticola* but not in other diplonemid species studied. However, each of the proteins encoded by these transcripts lacks a PTS. The authors thus concluded that the common ancestor of Euglenozoa had separate genes for OPRT and OMPDC that underwent duplications and fusions in different orders in the different progeny lineages, with targeting of the encoded bifunctional enzyme to glycosomes only in kinetoplastids.

Trypanosomatids, like many other protists including *Naegleria gruberi* are not capable of purine synthesis *de novo*, but rely on salvage from nucleotides (Supplementary Figure S3). The parasites have different isoforms for some enzymes of the purine-nucleotide cycle and the interconversion of purine bases and nucleotides. Previously, it was found that most but not all trypanosomatid enzymes involved in purine salvage, or in some cases one of the isoforms, possess a PTS1 and/or have been experimentally located in glycosomes: adenine phosphoribosyltransferase (APRT), hypoxanthine-guanine phosphoribosyl transferase (HGPRT), xanthine phosphoribosyl transferase (XPRT), inosine-5′-monophosphate dehydrogenase (IMPDH), guanosine monophosphate reductase, AMP deaminase and guanylate kinase (for *Leishmania* reviewed by Boitz et al., 2012). We detected orthologues of these enzymes with a PTS in the databases of most kinetoplastids, including the bodonids, and the diplonemids, but not in *Euglena* and *Naegleria* species (Supplementary Table S2), suggesting the pathway was compartmentalised at a very early stage of glycosome evolution. However, the compartmentalisation is partial, with variations evident in different species.

The two very different (22% identity) APRT isoforms in *T. brucei* have been studied in detail; APRT1 has no PTS and is cytosolic, whereas APRT2, with a PTS2, is glycosomal (Lüscher et al., 2013). However, only APRT1 displayed activity; no function could yet be attributed to APRT2 (Glockzin et al., 2022). It is not known whether APRT2 has activity in other euglenids. APRT2 possesses a PTS1 in other *Trypanosoma* species and in *P. confusum*, but it has no characteristic PTS in any other species analysed. Curiously, there is a specific XPRT not found in mammals and many other organisms present in Euglenozoa. In other protists, XPRT activity is often associated with HGPRT. Jardim et al. (1999) showed that, in *L. donovani*, the XPRT amino acid sequence is 33% identical to that of HGPRT with the HGPRT and XPRT genes arranged in tandem, pointing to gene duplication followed by evolution of one paralogue to a XPRT. The duplication possibly happened early in the evolution of the kinetoplastid lineage, because database analysis shows syntenic orthologues in all kinetoplastids. Only a single gene was detected in diplonemids and euglenids. The ancestral HGPRT may already have been glycosomal: a PTS was identified in most kinetoplastid HGPRTs, and also in diplonemids. XPRT has a PTS1 in all kinetoplastids studied.

Several of the glycosomal enzymes of pyrimidine biosynthesis and purine salvage use 5-phosphoribosyl-1-pyrophosphate (PRPP) as a substrate. Since this is a rather large molecule (390 Da), that may not easily traverse the glycosomal membrane, we looked for a glycosomal PRPP synthase. Proteomic analyses of *T. brucei* glycosomes suggested the presence of two isoenzymes, although both without a PTS (Güther et al., 2014). The two proteins, encoded by non-linked genes, are 54% identical and share significant similarity with an active PRPP synthase of *Leishmania donovani* of unspecified subcellular localization (Hendrickson et al., 1993). Orthologues of the two *T. brucei* candidate PRPP synthases can be detected in databases of all organisms analysed, but with a PTS in only a few kinetoplastid species (Supplementary Table S2).

### Bioinformatic chronology of glycosomal metabolism VI: Phosphatases

Although glycosomal proteins can be phosphorylated (Zhang et al., 2020), no protein kinases have been detected in trypanosomatid glycosomes. However, several phosphatases have been identified in *T. brucei* glycosomes (Szöőr et al., 2010; Güther et al., 2014). Phosphorylation of glycosomal proteins potentially occurs in the cytosol, prior to their import and, when appropriate, they are dephosphorylated by organellar phosphatases. Such a mechanism would probably imply metabolic regulation at a longer time span than usually is the case for enzyme activity modification by phosphorylation/dephosphorylation. It would link the phosphorylation/dephosphorylation process to the biogenesis of the organelles. Such mechanism is reminiscent of observations by Szöőr et al. (2019) made for the serine/threonine phosphatase PIP39 that possesses a PTS1. This phosphatase is itself phosphorylated outside the glycosomes by an as yet unidentified tyrosine kinase resulting in PIP39 activation and relocation to glycosomes. This occurs during the second stage of the process, triggered by a quorum-sensing mechanism, by which proliferating slender BSF trypanosomes first differentiate to quiescent stumpy BSF forms, which then develop further to procyclic-form (PCF) trypanosomes after ingestion by the tsetse fly. Intraglycosomal targets of PIP39 remain to be identified.

Since glycosomes are massively degraded by pexophagy during differentiation to PCFs and a new population of organelles is formed (Herman et al., 2008), it is feasible that the sequestering of PIP39 is meant to neutralise it after having exerted its activity in the cytosol upon the trigger for the second differentiation step. Yet, PIP39 with a PTS1 is also found in other kinetoplastids including the bodonids *T. borreli* and *B. saltans* which are parasitic and free living, respectively (Supplementary Table S2). Whether the protein is involved in performing a similar role in differentiation in all these other organisms when they must adapt to different environmental conditions remains to be studied.

Among the different types of protein phosphatases present in trypanosomatids are representatives of the ‘non-conventional’ ApaH-like phosphatases (Alphs) (Brenchley et al., 2007). One Alph has a PTS1 in *Trypanosoma* species, but no Alphs with a PTS were detected in any other trypanosomatid analysed. An Alph with a potential PTS2 was detected in the bodonid *T. borreli*, while different species of *Diplonema* possess an Alph with either a PTS2 or PTS1. The function of this glycosomal phosphatase is unknown, but it seems most likely that early in *Trypanosoma* evolution one Alph became rerouted to glycosomes.

A third glycosomal enzyme exhibiting phosphatase activity is one of the Nudix hydrolase isoforms found in *T. brucei. T. brucei* and *T. cruzi* contain inorganic polyphosphate (polyP) in different cell compartments including acidocalcisomes, nucleus, cytosol and glycosomes (Negreiros et al., 2018). The role of polyP in glycosomes is not yet clear: it is not known how it arrives in glycosomes or whether it is synthesized inside glycosomes. It could be a storage product of Pi or PPi, or have a structural function.

The glycosomal Nudix hydrolase shows exo- and endophosphatase activity towards polyP (Cordeiro et al., 2019). The enzyme has a PTS1, and genes encoding orthologues with a PTS1 are also found in various other trypanosomatids but were not found in other euglenozoans. It remains to be determined if glycosomes in other species also contain polyP and if the polyP hydrolysis is the only or main activity of the enzyme.

### Bioinformatic chronology of glycosomal metabolism VII: Glycosomal ABC transporters (GATs).

Peroxisomal membranes usually contain multiple ABC transporters of the D-subfamily-type. These are half-size transporters with typically one transmembrane domain (TMD) comprising six transmembrane-spanning segments and one cytosol-facing C-terminal nucleotide-binding domain (NBD) with canonical Walker A and B motifs for ATP binding (Chornyi et al., 2021). They form functional transporters with a dimeric NBD by homo- or heterodimerisation. Human and *S. cerevisiae* peroxisomal ABC transporters import fatty acids and/or acyl-CoAs. Three half-size ABC transporters with glycosomal localisation occur in *T. brucei*, called GAT1-3 (for glycosomal ABC transporter) (Yernaux et al., 2006). GAT1 and GAT2 are ∼30% identical and display ∼25% identity with human, yeast and plant homologues. GAT3 is very different. GAT1 mediates uptake of oleoyl-CoA into *T. brucei* glycosomes (Igoillo-Esteve et al., 2011). Specificities of GAT2 and GAT3 require determination.

Sequences homologous with each *T. brucei* GAT were detected in all euglenozoans and *N. gruberi* (Supplementary Figures S4A–C). Between different kinetoplastid genera, pairwise identities vary between 50 and 60% for each of the GATs from *T. brucei* and *T. cruzi*, between 50 and 85% between the GATs from different Leishmaniinae genera and values down to 10–30% when comparing the corresponding proteins from more distantly related euglenozoans or these with three different predicted *Naegleria* peroxisomal transporters. Supplementary Figure S4D shows a multiple alignment of GAT1, two and three from each TriTryps species plus two well-characterised half-size *S. cerevisiae* ABC transporters, Pxa1p and Pxa2p. The six transmembrane segments that together form the TMD in the N-terminal half are readily identifiable in all sequences, as well as the C-terminal NBD domain, including the Walker A and B motifs and the conserved linker that forms the so-called ABC signature. Together, the data indicate three different half-size ABC transporters were present in the common euglenozoan ancestor.

### Bioinformatic consideration of glycosome assembly: Peroxin evolution in Euglenozoa

Glycosome biogenesis occurs by a process similar to that of peroxisomes in mammalian, plant and yeast cells, involving conserved proteins called peroxins or PEX proteins. *T. brucei* peroxins acting in various aspects of biogenesis have been identified–and orthologues also in other trypanosomatids (Supplementary Figures S5) (reviewed by Galland and Michels, 2010; Bauer and Morris, 2017; Kalel and Erdmann, 2018; Michels and Gualdrón-López, 2022).

**FIGURE 6:**
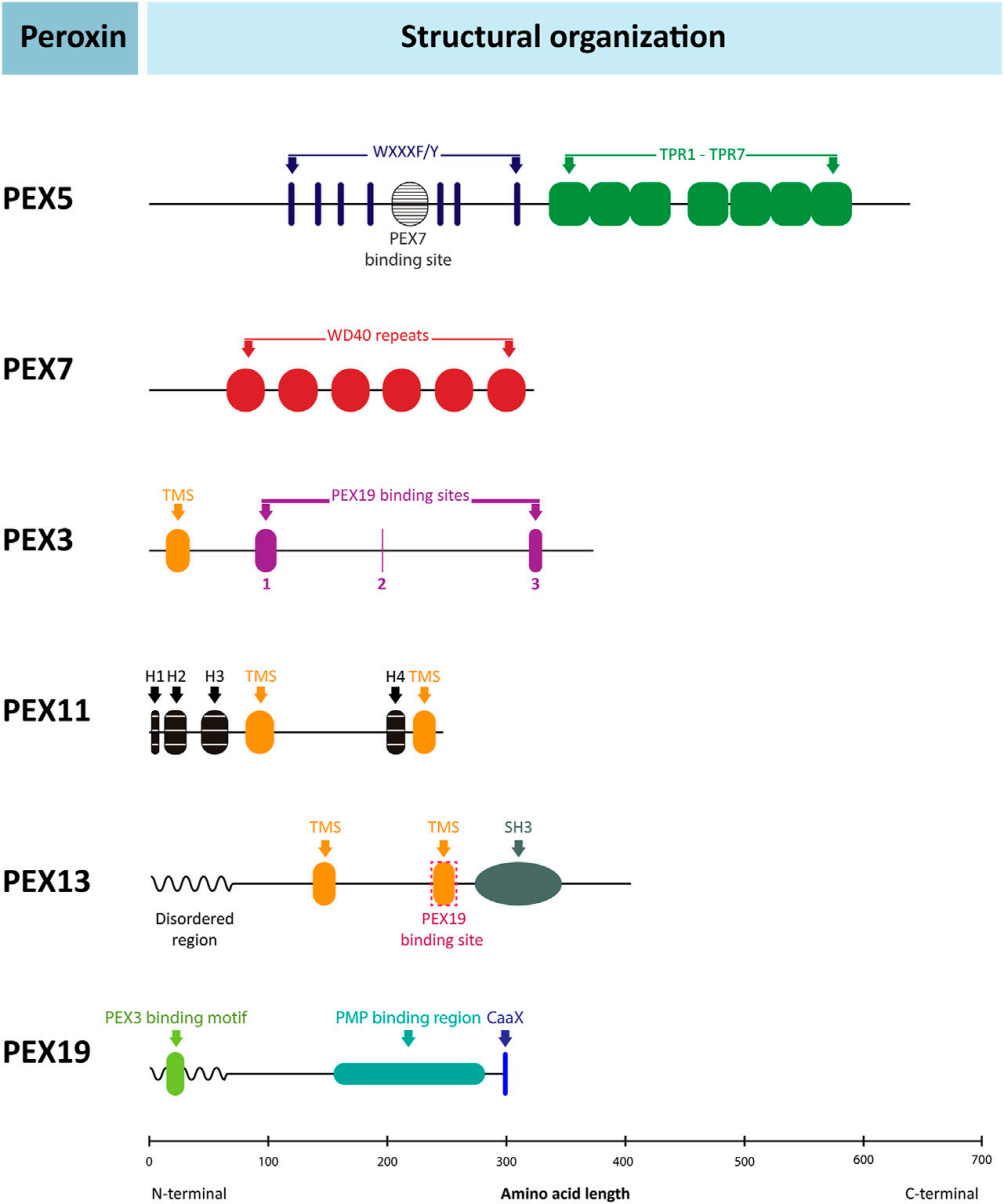
Structural organisation of peroxins. Schematic representation of the structure of several human peroxins with the relative position of their functional motifs indicated by coloured boxes and identities specified. The organisation of the corresponding peroxins from yeasts and plants is very similar, but many important differences are found in the peroxins of Euglenozoa as discussed in the text and shown in detail in the sequence alignments in Figure 7 and Supplementary Figures S6–S12. The human peroxins IDs are: PEX5 (P50542); PEX7 (O00628); PEX3 (P56589); PEX11α (O75192); PEX13 (Q92968); PEX19 (P40855). TPR: tetratricopeptide repeats; TMS: transmembrane segment; H1-3: amphiphilic helices.

In general, trypanosomatid peroxins are noticeably divergent from their peroxisomal counterparts in mammals, plants and yeasts; in some cases, homology is barely recognisable. Key questions are therefore whether this divergence is a characteristic of all euglenozoans; whether divergence is evident in only some euglenozoan lineages, perhaps coincident with the shift from typical peroxisome to glycosome; or whether peroxin divergence is seen in other Discoba, e.g. *Naegleria* spp. Using bioinformatics, we addressed these questions for different peroxins, such as PEX5 and PEX7, and others which in previous *T. brucei* research showed particularly unique features compared to counterparts in peroxisomes of mammals, yeasts and plants (i.e., PEX3, PEX13, PEX19, PEX11/GIM5A and GIM5B). The general structural organisation of these peroxins is presented in Figure 6.

**PEX5** is a cytosolic protein composed of two domains: an N-terminal domain responsible for association with the docking complex in the peroxisomal/glycosomal membrane and a C-terminal PTS1-protein binding domain. The N-terminal domain contains several WxxxF/Y pentapeptide motifs with the propensity to form amphiphilic helices that mediate binding of PEX5 to PEX14. A species-dependent variable number of pentapeptide motifs has been observed, from two in *S. cerevisiae* to eleven in some plants; three were identified in *T. brucei*, with boxes 1 and 3 showing high binding affinity for PEX14 (Choe et al., 2003). In mammals and plants, PTS2-protein import has been shown to converge with a PTS1-protein import route by interaction of PEX7 with an approximately 35-residues long region in the N-terminal half of PEX5 called PEX7-binding box (Braverman et al., 1998; Otera et al., 1998). In contrast, in *S. cerevisiae* PTS1- and PTS2-protein import occurs via separate routes (Montilla-Martinez et al., 2015). A sequence similar to the PEX7-binding box of mammalian PEX5 has been found in PEX7-binding proteins of yeasts and fungi (Dodt et al., 2001; Einwachter et al., 2001) as well as in PEX5 of the TriTryps species with experimental data supporting an interaction (Galland et al., 2007). The C-terminal half of PEX5 is a tetratricopeptide repeat (TPR) domain, consisting of seven degenerate TPR motifs. The crystal structure of the PEX5 TPR domain has been solved for human and *T. brucei* (Gatto et al., 2000; Sampathkumar et al., 2008). The repeats form two subdomains, made up of TPR1-3 and TPR5-7 respectively, connected by TPR4, thus forming a cavity in which the C-terminal peptide of a PTS1 protein binds. The seven TPR motifs of the C-terminal domain are found in all euglenozoans. The positions of the three pentapeptide motifs in the N-terminal domain are also well conserved, although *B. ayalai* has lost the first and in *P. confusum* the second appears absent (Supplementary Figure S6). The putative PEX7-binding box is found in all kinetoplastids, but its presence in diplonemids, *E. gracilis* and *N. fowleri* is less certain.

**PEX7** is a cytosolic protein containing six WD repeats that make up almost the entire length of the protein. Each repeat has about 40–60 residues with a characteristic central tryptophan-aspartate (WD) pair. The protein forms a ring structure with a seven-bladed β-propeller fold. Six blades are formed by each of the WD repeats and most of the seventh by the N-terminal region (although it has low similarity to WD motifs) that unites with the C-terminal end to close the ring (Pan et al., 2013; Kunze, 2020). The PTS2 nonapeptide forms an amphipathic α-helix that binds in a horizontal, large cleft on top of PEX7.

The WD repeats that form six of the seven blades of the PEX7 propeller structure are recognisable in a multiple sequence alignment of all euglenozoan PEX7s, although the primary structure is highly variable (Supplementary Figure S7).

**FIGURE 7:**
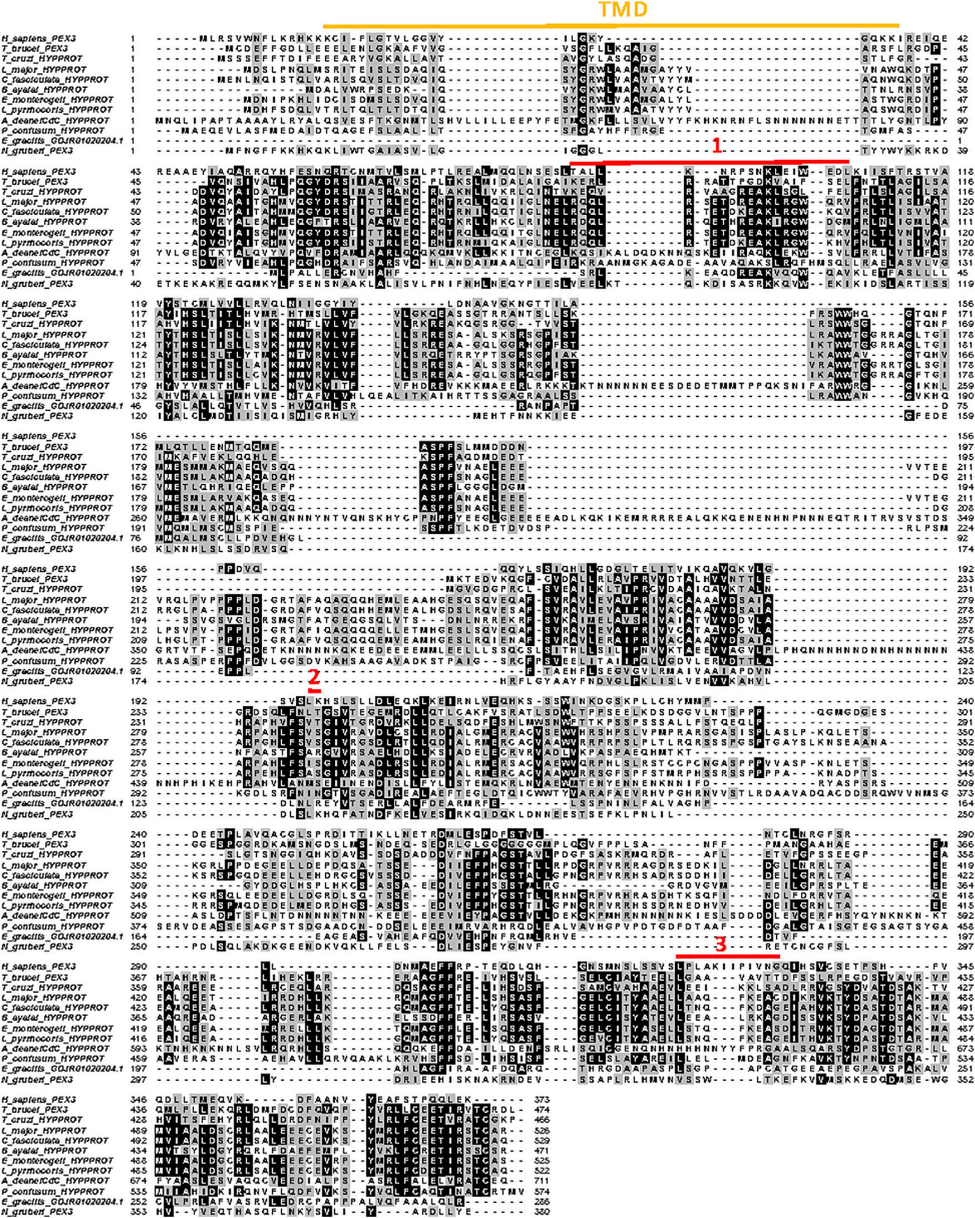
Alignment of PEX3 amino-acid sequences from selected Euglenozoa, *Naegleria* and human. Sequence IDs: *H. sapiens*: P56589; *T. brucei*: Tb927.11.10260; *T. cruzi*: TcCLB.510,719.280*; L. major*: LmjF.36.4010; *C. fasciculata*: CFAC1_280044800; *B. ayalai*: Baya_134_0190; *E. monterogeii*: EMOLV88_360046900; *L. pyrrhocoris*: LpyrH10_06_1720; *P. confusum*: PCON_0042250; *E. gracilis*: GDJR01020204.1; *N. gruberi*: NAEGRDRAFT_68465. The transmembrane region is highlighted by the yellow line above the alignment, and the amino acids involved in PEX19 binding are indicated by red lines numbered 1 to 3 (Kalel et al., 2019). The alignment was made in MUSCLE.

**PEX3** is a central component of the machinery for insertion of proteins into the peroxisomal membrane and considered a master regulator of peroxisome biogenesis. It is an integral membrane protein with a single transmembrane segment close to its N-terminus and three PEX19 binding sites. Its presence in glycosomal membranes of trypanosomes remained enigmatic for a long time. Only recently, two groups independently identified the *T. brucei* peroxin using different bespoke methods. Banerjee et al. (2019) found a candidate PEX3 using the HHpred protein comparative tool for remote protein homology detection and structure prediction, while Kalel et al. (2019) used a combinatory approach involving identifying PEX19-interacting proteins and secondary structure homology. The teams subsequently confirmed PEX3 candidature through functional studies. They reported very low overall sequence identity between the *T. brucei* protein and known peroxisomal PEX3s (<7%, or 11% if large trypanosome-sequence specific insertions are omitted), but higher (>30%) for the known PEX19 binding sites. The very high diversity of PEX3s complicated their identification in the Euglenozoa, even with knowledge of the *T. brucei* peroxin, but a multiple alignment of selected putative PEX3 sequences is presented in Figure 7. It shows that also amongst most of these protists the degree of its conservation is very low, although 65–80% identity is found between the PEX3s of the Leishmaniinae genera; for *T. cruzi*, only an unlikely PEX3 candidate with an estimated 12% identity to the *T. brucei* PEX3 was found. For the residues involved in PEX19 binding, as reported for *T. brucei* PEX3 (Kalel et al., 2019), some conservation is generally found.

**The PEX11 family**, which in most eukaryotes where peroxisome biogenesis has been studied is a multigene family arising as consequences of lineage-specific duplications and paralogue divergence (Schrader et al., 2012; Schrader et al., 2016; Jansen et al., 2021). PEX11 isoforms are integral membrane proteins, although for *S. cerevisiae* peripheral association with the membrane was reported. The N- and C-termini are present in the cytosol, but detailed information about the topology is limited. Usually two or four transmembrane-spanning segments are predicted from the sequences, two for *T. brucei* PEX11 (Lorenz et al., 1998). PEX11s are involved in regulating peroxisome size and number. In mammals, PEX11β functions in peroxisome elongation and recruiting proteins necessary for constriction and fission of the organelles. The specific molecular roles of PEX11α and γ in this process are less known; they don’t complement loss of PEX11β. Additionally, PEX11 of yeast has been shown to also possess the capacity to form pores in the peroxisomal membrane allowing passage of solutes with molecular mass up to 300–400 Da (Minthoff et al., 2016). Trypanosomes also contain three family members, called PEX11, GIM5A and GIM5B. Their PEX11 seems to exert a role most similar to that of mammalian PEX11β, since overexpression in *T. brucei* resulted in changing normally spherical glycosomes into clusters of long tubules, whereas knockdown led to fewer but larger organelles (Lorenz et al., 1998). The roles of GIM5A and GIM5B are less clear. The genes are tandemly arranged, and the encoded proteins are 88% identical, but very different (21% identity) from PEX11 of which the gene is present on a different chromosome. GIM5A/B depletion resulted in a pleiomorphic phenotype, demonstrating the two proteins have non-redundant but overlapping functions. Changes included decreased glycosome numbers and alterations in their morphology, as well as increased cellular fragility and several metabolic effects, some suggestive that like PEX11 in yeast these proteins may also be involved in metabolite transport (Voncken et al., 2003).

PEX11 sequences were identified from different Euglenozoa (Supplementary Figure S1). Despite the high diversity, the positions of two predicted transmembrane segments appear conserved in the different PEX11 candidates. Conserved are three peptide sequences in the N-terminal region with propensity to form amphipathic helices that, in *Penicillium chrysogenum*, are essential for the membrane remodelling capacity of PEX11 (Opalinski et al., 2011; Schrader et al., 2012). An amphipathic helix near the C-terminus is also conserved, but its function is still unknown (Zientara-Rytter et al., 2022).

Sequences highly related to *T. brucei* GIM5A/B were detected in all kinetoplastids and diplonemids analysed (a selection is aligned in Supplementary Figure S9), but not in euglenids (although a possible GIM5-related fragment was found for *Rhabdomonas*) or *Naegleria*. Analogous to *T. brucei*, in several other kinetoplastids two linked, related GIM5 genes were detected, indicating the gene duplication characterised by Voncken et al. (2003) was most likely not a recent event.

**PEX13** is present in all members of the organelle family of peroxisomes, including glycosomes. This protein is part of the docking complex for the cytosolic receptor charged with a matrix protein to be imported. It is an integral membrane protein with N- and C-terminus exposed to the cytosol. Characteristic for PEX13 is an N-terminal part containing a proline-rich region, often with a KWPE motif, followed by a glycine-rich segment with many Tyr-Gly motifs. The C-terminal half contains two membrane-spanning helices, followed by a SH3 domain in the peroxin of yeasts and animal; this domain is absent from plant PEX13. Curiously, in trypanosomatid glycosomes two PEX13 isoforms, PEX13.1 and PEX13.2, are present; strikingly, both share very low sequence identity with the single PEX13 identified in peroxisomes of other taxa (7–18% identity). They also differ markedly between each other (11–16% identity) (Verplaetse et al., 2009; Brennand et al., 2012; Verplaetse et al., 2012). PEX13.1 contains the Tyr-Gly motifs and SH3 domain, but is unusual because it lacks the proline-rich region near the N-terminus and has a typical PTS1-like motif (-TKL, conserved in the TriTryps parasites) at its cytosolically exposed C-terminus, not found in any other PEX13. In contrast, PEX13.2 possesses a proline-rich region with a RPWE motif and a Tyr-Gly motif containing glycine-rich segment, but no SH3 domain. Both PEX13 proteins were located in the *T. brucei* docking complex, where they interact with each other and with PEX14 (Verplaetse et al., 2012; Crowe et al., 2020).

PEX13.1 sequences were detected in the databases for representatives of all kinetoplastid groups, but not for diplonemids and euglenids (Supplementary Figure S10). The unique features detected in the *T. brucei* peroxin are generally present, despite high sequence diversity. In contrast, potential PEX13.2 sequences were found for kinetoplastids and euglenids (*E. gracilis*, *E. longa* and *R. costata*) (Supplementary Figure S11). Like PEX13.1, characteristic features identified previously for the *T. brucei* peroxin are conserved despite high sequence variation. Together, the data indicate this peroxin was realistically already present in the common ancestor of the Euglenozoa. It remains to be determined when this protein originated. We did not detect a homologue in the databases of *Naegleria* species.

**PEX19** is a cytosolic peroxin that functions as receptor and chaperone of peroxisomal membrane proteins (PMPs). PEX19, complexed with a PMP synthesised in the cytosol, docks at the peroxisomal membrane at PEX3, followed by membrane insertion. The N-terminal part of PEX19 contains binding sites for PEX3 and/or PEX14, whereas PMP binding occurs at the C-terminal domain. Furthermore, PEX19 proteins previously studied contain a CaaX motif for farnesylation near the C-terminus. Farnesylation is required for PEX19 structural integrity and its recognition of PMPs (Rucktäschel et al., 2009; Emmanouilidis et al., 2017). Curiously, trypanosomatid PEX19 lacks the motif for posttranslational modification that plays such an important role in peroxisome biogenesis in other organisms (Banerjee et al., 2005; Yernaux et al., 2006).

Putative PEX19 sequences were detected in most kinetoplastids and some euglenids and *Naegleria* species, but not in diplonemids (Supplementary Figure S12). These PEX19 candidates show considerable sequence variation. Importantly, the C-terminal CaaX farnesylation motif is absent in all euglenozoans, but is present in PEX19 from *Naegleria*. How euglenozoan PEX19 functions without apparent farnesylation remains to be determined.

## Discussion: Reconciling bioinformatics observations with a ‘how’ and ‘why’ of glycosome evolution

Combining literature survey with bioinformatic predictions of presence or absence of PTS motifs, we refine a timeline for how metabolically complex glycosomes evolved in Euglenozoa. Availability of much sequence data for diverse discoban protists allows us to add considerable depth to a timeline offered by Gualdrón-López et al. (2012a). Recently, Durrani et al. (2020) reported a similar global bioinformatics approach. Caveats associated with estimating peroxisome localisation on a basis of detecting PTS motifs must be acknowledged: many peroxisomal and glycosomal proteins do not possess consensus PTS motifs; proteins with a PTS are often not detected in these organelles but elsewhere in a cell; and Güther et al. (2014), analysing the high-confidence proteome of epitope-tagged enriched glycosomes of PCF *T. brucei*, concluded use of PTS sequence searches in *T. brucei* has a sensitivity of <40% and a specificity of <50%.

In part, the efficiency with which PTS1 motifs are recognised as determinants for peroxisome targeting is influenced by the identity of amino acid residues immediately adjacent to a PTS motif (Brocard and Hartig, 2006). Yet, the frequency of dual protein localisation within peroxisomes and cytosol in many eukaryotes together with the wide range of PTS motifs known to confer organelle targeting (Sommer et al., 1992; Freitag et al., 2018; Kunze, 2020), often in heterologous systems, argue strongly for the value inherent in estimating peroxisome localisation from PTS motif conservation in related eukaryotes. Indeed, minimal simplicity of C-terminal tripeptide or N-terminal nonapeptide motifs, often without absolute requirement for amino acid specificity in order to confer some degree of organelle targeting, is readily compatible with Martin’s view that metabolic pathways could easily be rerouted in organisms whenever inevitable minor mis-targeting of proteins provides a small selective advantage; protein mis-targeting happens often in eukaryotic cells (Martin, 2010). Conversely, free passage of metabolites <400 Da across glycosomal membranes (Quiñones et al., 2020; Michels and Gualdrón-López, 2022) suggests ‘hard-wiring’ organelle re-routed cytosolic pathways is not necessarily readily achieved, thereby explaining a sometimes patchwork conservation of PTS motifs observed in some euglenozoan enzymes, as indicated in Supplementary Tables S1 and S2. In this work, we also dated divergence of trypanosomatid peroxins to an early point in euglenozoan evolution. Provocatively, divergence of a broadly conserved peroxisome biogenesis machinery may have helped cement transitions from ‘mis-targeting’ to ‘re-compartmentalisation’ for enzymes and processes that with rare exception are cytosolic in other eukaryotes.

Possible selective advantage(s) obtained by sequestering glycolytic enzymes within peroxisomes has often been addressed since the serendipitous discovery of glycosomes in experimentally tractable tropical disease parasites during the 1970s. Initially, compartmentalisation was considered to sustain high glycolytic flux (Borst, 1986; Opperdoes, 1987; Borst, 1989; Michels and Opperdoes, 1991) or physically separate opposing pathways as an alternative to activity regulation, thereby avoiding futile cycling (Michels and Hannaert, 1994). Such hypotheses have been discarded (reviewed by Gualdrón-López et al., 2012a), but a role for pexophagy–the turnover ‘en masse’ of whole peroxisomes enabling rapid, extensive metabolic reprogramming–remains highly relevant to theorizing about glycosome origin and evolution. We argued earlier in this work that free-living ancestral euglenozoans plausibly lived in dynamic conditions where ability to turnover a major part of carbohydrate metabolic network quickly conceivably provided key selective advantage. Moreover, massive glycosome turnover involving pexophagy occurs during differentiation of extant trypanosomatids and is potentially key to their success as parasites (Herman et al., 2008; Cull et al., 2014).

**FIGURE 8:**
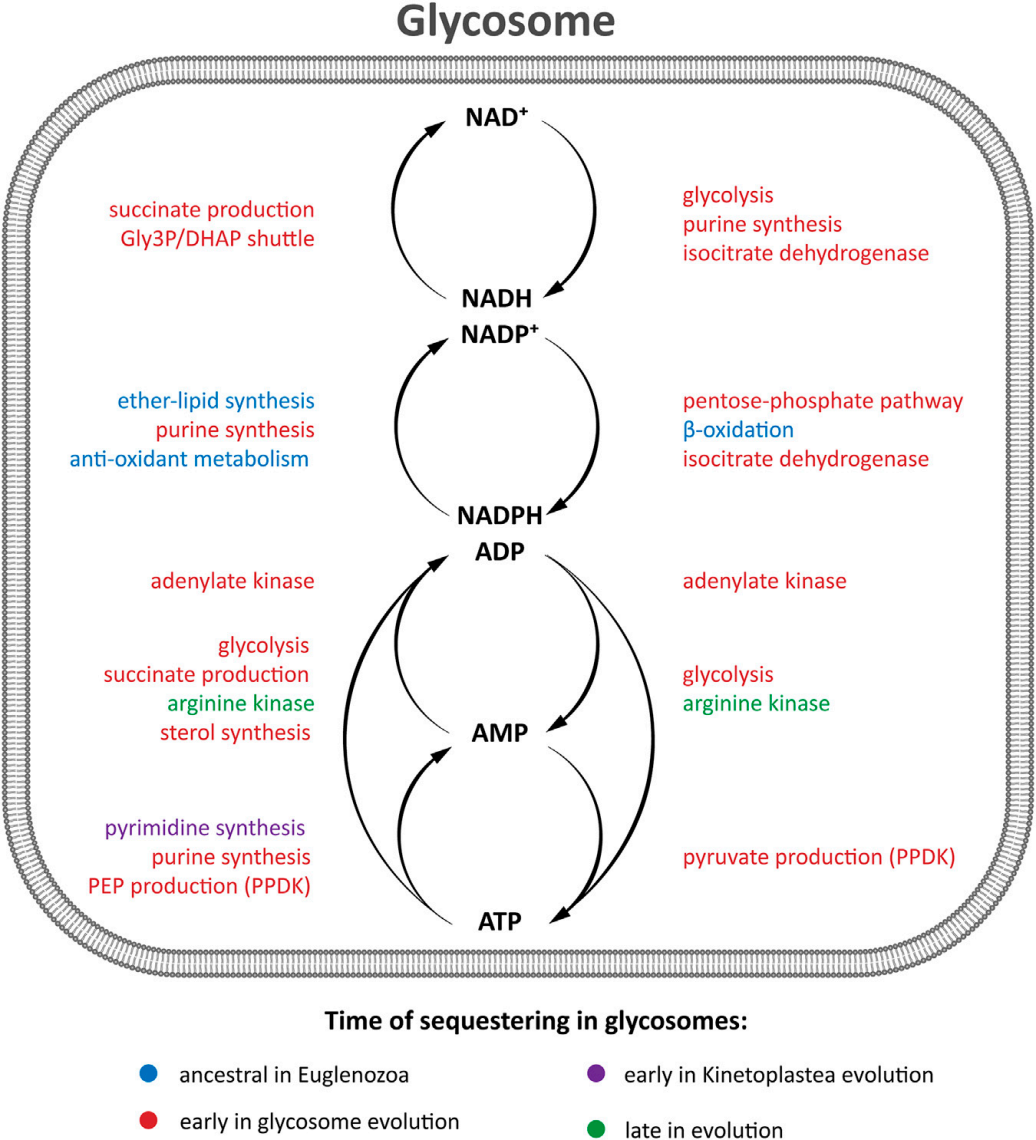
Glycosomal homeostasis of cofactors. Glycosomes harbour a pool of adenine nucleotides and nicotinamide-adenine nucleotides, separate from the cytosolic pool, that is involved in various branches of glycosomal catabolism and anabolism. The presence or absence of PTS motifs suggests that enzymes of the metabolic processes listed in the figure were sequestered at different stages in the evolution of glycosomes: already present in peroxisomes of ancestral organisms (blue); sequestered within glycosomes during or shortly after the origin of these organelles from peroxisomes (red); sequestered only in kinetoplastids at an early stage (mauve); sequestered late in glycosomes of some taxa (green). However, it should be noted that specific enzymes of some processes mentioned in the figure were not compartmentalised or even lost from all or certain taxa, as described in the text.

Besides endowing cells efficient adaptation to changing conditions, development of glycosomes may have provided other or additional selective advantage. They contain enzymes to catalyse reactions involving ATP, NADH, and NADPH (Figure 8). Strikingly, use and production of each of these cofactors seems in balance within glycosomes (Opperdoes and Borst, 1977; Bakker et al., 1997); the membrane is not permeable for these bulky molecules and there are no indications for their transport across the membrane at a rate commensurate with the metabolic fluxes through the organelles. Glycosomes thus create separate compartments with their own redox state (ratios of NADH/NAD^+^ and NADPH/NADP^+^) and ATP/ADP ratio different from that in cytosol and mitochondrion, thus providing the cell more metabolic flexibility. Similar reasoning has been offered to explain the unexpected dual localisation in peroxisome and cytosol of enzymes from the central part of glycolysis in several fungi; here, iso-enzyme targeting is achieved through cryptic PTS motifs made available from alternative splicing or translation stop-codon read-through of RNA encoding cytosolic triose-phosphate isomerase, GAPDH, and/or PGK (Freitag et al., 2012). Of course, crosstalk occurs between different compartments by the exchange of metabolites–including substrates and products of enzymes using the cofactors, but the redox state and ATP/ADP ratios will differ as demonstrated *in silico* for *T. brucei* using realistic kinetic models (Bakker et al., 1997; Bakker et al., 2000).

A major revelation from the *in silico* analysis we report here is that much of the metabolic complexity evident in trypanosomatid glycosomes was already established prior to the divergence of a last common ancestor of diplonemids and kinetoplastids. During subsequent evolution of both lineages only relatively minor embellishments and alterations to this complexity occurred (Figure 8). Strikingly, H_2_O_2_ metabolism in glycosomes also appears more prominent than previously thought. Atypical localisation of ubiquitous cytosolic enzymes to peroxisomes is not evident in either euglenids or the closest distant relations of the Euglenozoa for which relevant genome data is available (*Naegleria* spp.).

Looking forwards, gene and candidate PTS motif inventories archived in Supplementary Tables S1 and S2 provide foundations for experimental interrogation of evolutionary scenarios we have discussed. Critically, our interpretation of the data is compatible with two phenomena we consider key in glycosome evolution: turnover of organelles by pexophagy and permeability of peroxisome and glycosome membranes to metabolites <400 Da. Finally, parallels and divergence between glycosome evolution and the establishment of different atypical peroxisomes in other eukaryotes–*e.g.* peroxisomes in methylotrophic yeasts, woronin bodies in filamentous fungi, glyoxysomes in plants, recently discovered anaerobic peroxisomes in anaerobic amoebae (Le et al., 2020; Verner et al., 2021) – provide other avenues for further discussion and experimentation. Many outstanding questions about glycosomes have recently been discussed by Michels and Gualdrón-López (2022). Additional questions that speak to the evolution of these organelles and could be addressed experimentally are listed in Table 1.

**TABLE 1:** Outstanding questions about glycosome evolution.

• Do free-living kinetoplastids and diplonemids alter their glycosomal enzyme content when they encounter environmental changes? If so, then does pexophagy play a role?
• Why is PFK evolution so complex in Euglenozoa? What was (were) the evolutionary driver(s) for acquisition and losses of glycosomal PFKs with different phospho donor specificity (i.e. ATP versus PPi)?
• What explains the isoform complexity of PFK families in different diplonemids?
• Is an acyl-CoA oxidase or acyl-CoA dehydrogenase involved in glycosomal fatty-acid β-oxidation? Has use of these enzymes swapped around during glycosome evolution?
• Looking beyond proposed ecological advantages, did peroxin divergence or another biochemical trait influence large-scale re-compartmentalisation of carbohydrate metabolism in diplonemids and kinetoplastids?
• Why did the very divergent PEX13.2 paralogue evolve?
• Why is absence of PEX19 farnesylation not a problem for glycosome biogenesis or integrity?
• What evolutionary and functional advantages are provided by two highly divergent members of the PEX11 family, GIM5A and GIM5B?
• Does glycosomal master regulator PIP39 play a role in the developmental differentiation of other euglenozoans? How did its role in trypanosome differentiation evolve?
• Are peroxisomes or glycosomes present in Symbiotida, the enigmatic, fourth lineage of Euglenozoa?

## Data Availability

The original contributions presented in the study are included in the article/Supplementary Material, further inquiries can be directed to the corresponding author.

## Footnotes

1 Note: Diplonemid diversity has only begun to be truly recognised during the last 12–15 years. Consequently, taxonomic reassignment should see reclassification of *Diplonema papillatum* to *Paradiplonema papillatum*, separate from other species in the genus *Diplonema* (Tahyreva et al., 2022). Here, we use nomenclature still in use.
