# Supplementary material for: Delineating transitions during the evolution of specialised peroxisomes: Glycosome formation in kinetoplastid and diplonemid protists
Source: Front Cell Dev Biol. 2022 Sep 12;10:979269. doi: 10.3389/fcell.2022.979269 (PMC9512073; doi:10.3389/fcell.2022.979269)
Supplement: Supplementary file 2 [file DataSheet1.PDF]

Supplementary Material

Legend Supplementary Tables 1 and 2

Orthologues of *Trypanosoma brucei*, *Trypanosoma cruzi* and/or *Leishmania* spp. ('TriTryps') proteins as detected in databases of other, selected Euglenozoa species and *Naegleria* spp. Searches were made in the databases using as queries sequences of proteins that have previously experimentally been located in glycosomes. Positive hits were analyzed for the presence or absence of peroxisomal-targeting signals (PTS) as described in the accompanying paper. Potential PTS1 and/or PTS2 motifs detected in the orthologous proteins are specified in the tables, whereas the lack of a consensus PTS motif is indicated by a hyphen. It is mentioned when no orthologues were detected (either because they may be absent from the organism, or the database is still incomplete).

Files: Supplementary_Table_1.xlsx and Supplementary_Table_2.xlsx

Supplementary Figures

Supplementary figures 1 – 3 provide the metabolic context of the Euglenozoan enzymes analysed in the accompanying paper. Each of these figures, and Figures 2 and 5 in the main paper, present a scheme of a distinct part of glycosomal metabolism in trypanosomes. Enzymes containing a type 1 or type 2 peroxisomal targeting signal (PTS1 or PTS2) are indicated. However, many enzymes in the schemes don't have a PTS and many of the enzymes are not necessarily present in glycosomes. Indeed, many of them have not been detected in proteomics analyses of glycosomes (Colascante et al., 2006; Vertommen et al., 2008; Colasante et al., 2013; Güther et al., 2014; Acosta et al., 2019). Nonetheless, the presence of glycosomal membrane proteins forming non-selective pores allowing permeation of solutes with a Mr of approximately 300-400 Da (thus most metabolites of intermediary metabolism) enables fluxes through pathways with enzymes distributed on both sides of the membrane, in the glycosomal matrix and the cytosol (Gualdrón-López et al., 2012; Quiñones et al., 2020; Michels and Gualdrón-López, 2022). Designated transporters are required for bulky compounds such as fatty acids/acyl-CoAs and nucleotide sugars.

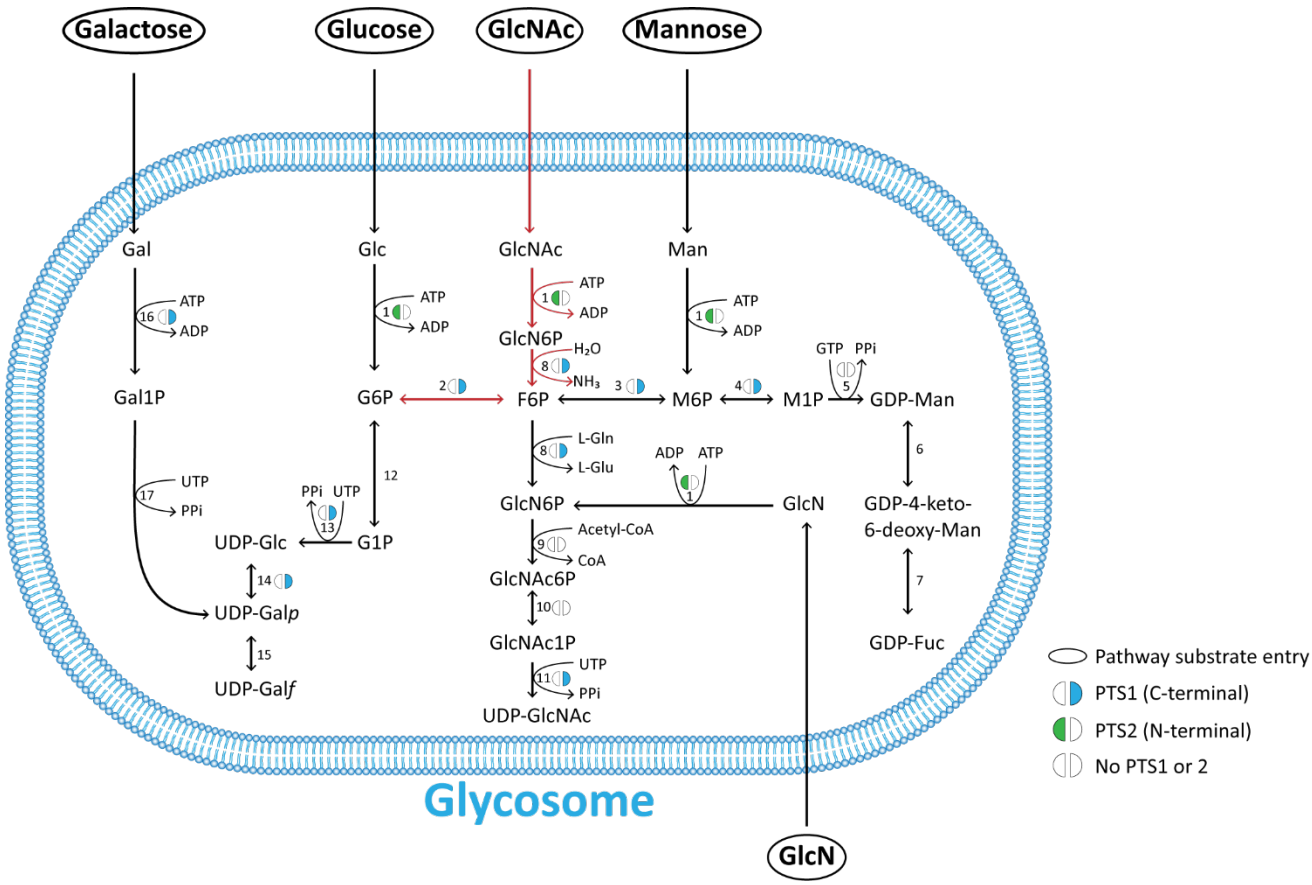

Supplementary Figure 1. Glycosomal nucleotide-sugar biosynthesis and galactose metabolism. Enzymes: **1**. hexokinase; **2**. glucose-6-phosphate isomerase; **3**. mannose-6-phosphate isomerase; **4**. phosphomannomutase; **5**. mannose-1-phosphate guanyltransferase; **6.** GDP-mannose 4,6-dehydratase; **7**. GDP-L-fucose synthetase **8**. glucosamine-6-phosphate deaminase; **9**. glucosamine-6-phosphate N-acetyltransferase; **10**. phosphoacetylglucosamine mutase; **11**. UDP-N-acetylglucosamine pyrophosphorylase; **12**. phosphoglucomutase; **13**. UDP-glucose pyrophosphorylase; **14**. UDP-galactose 4'-epimerase; **15**. UDP-galactopyranose mutase; **16**. galactokinase; **17**. UDP-hexose pyrophosphorylase. Red arrows show the pathway in intracellular amastigotes of *L. major* (Naderer et al., 2010). Metabolites: **G6P**: glucose 6-phosphate; **G1P**: glucose 1-phosphate; **F6P**: fructose 6-phosphate; **M6P**: mannose 6-phosphate; **M1P**: mannose 1-phosphate; **GDP-Man**: GDP-mannose; **Gal**: galactose; **Gal1P**: galactose 1-phosphate; **GlcN6P**: glucosamine 6-phosphate; **GlcNAc6P**: N-acetyl-glucosamine 6-phosphate; **GlcNAc1P**: N-acetyl-glucosamine 1-phosphate; **UDP-GlcNAc**: UDP-N-acetyl-glucosamine; **UDP-Glc**: UDP-glucose; **UDP-Gal*p***: UDP-galactopyranose; **UDP-Gal*f***: UDP-galactofuranose. **PPi**: Inorganic pyrophosphate; **UTP**: Uridine triphosphate; GDP-Fuc: GDP-fucose.

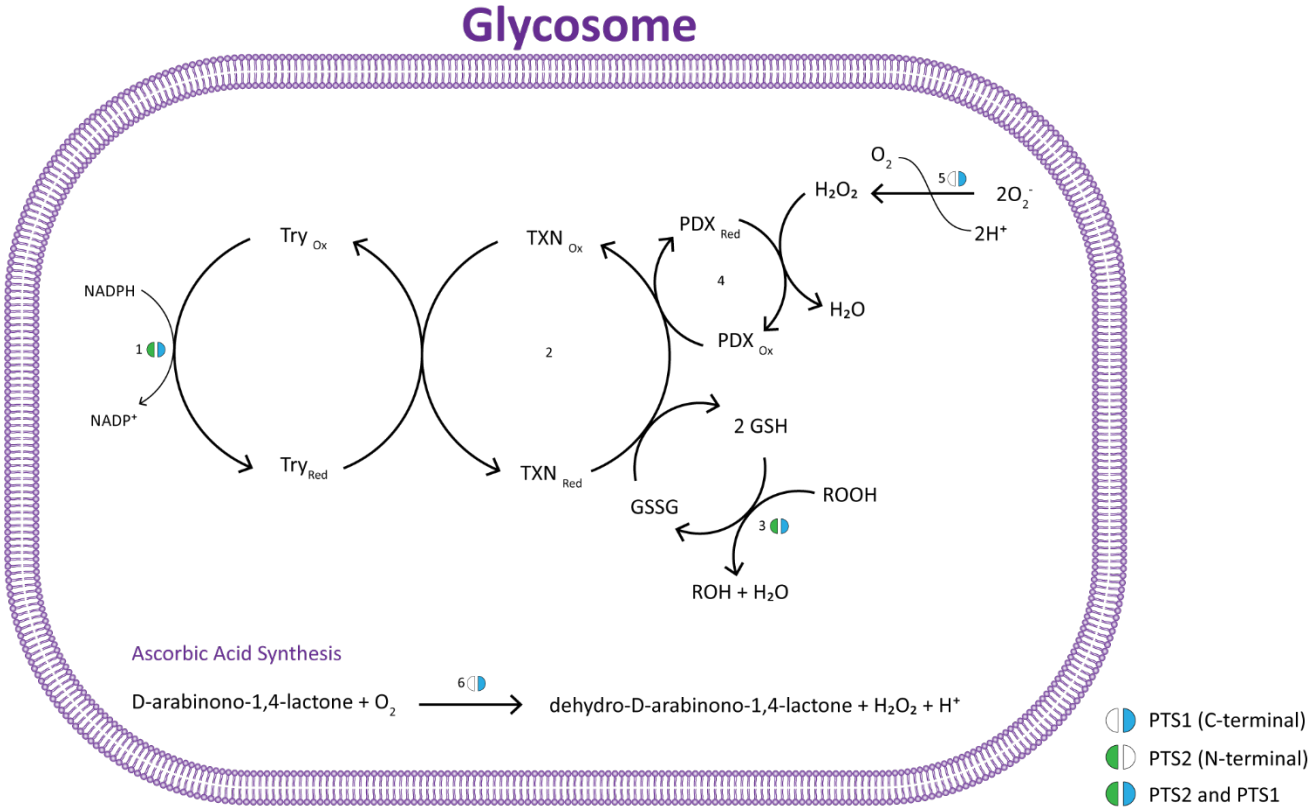

Supplementary Figure 2. Antioxidant metabolism. Enzymes: **1.** trypanothione reductase **2**. tryparedoxin; **3.** glutathione peroxidase-like protein; **4**. peroxiredoxin; **5**. iron-dependent superoxide dismutase; **6**. L-galactonolactone oxidase. **Try**: trypanothione; **TXN**: tryparedoxin; **PDX**: peroxiredoxin; **GSSG**: glutathione disulphide; **GSH**: glutathione. For a detailed description of the trypanothione-dependent antioxidant system in trypanosomes, see Leroux and Krauth-Siegel (2016).

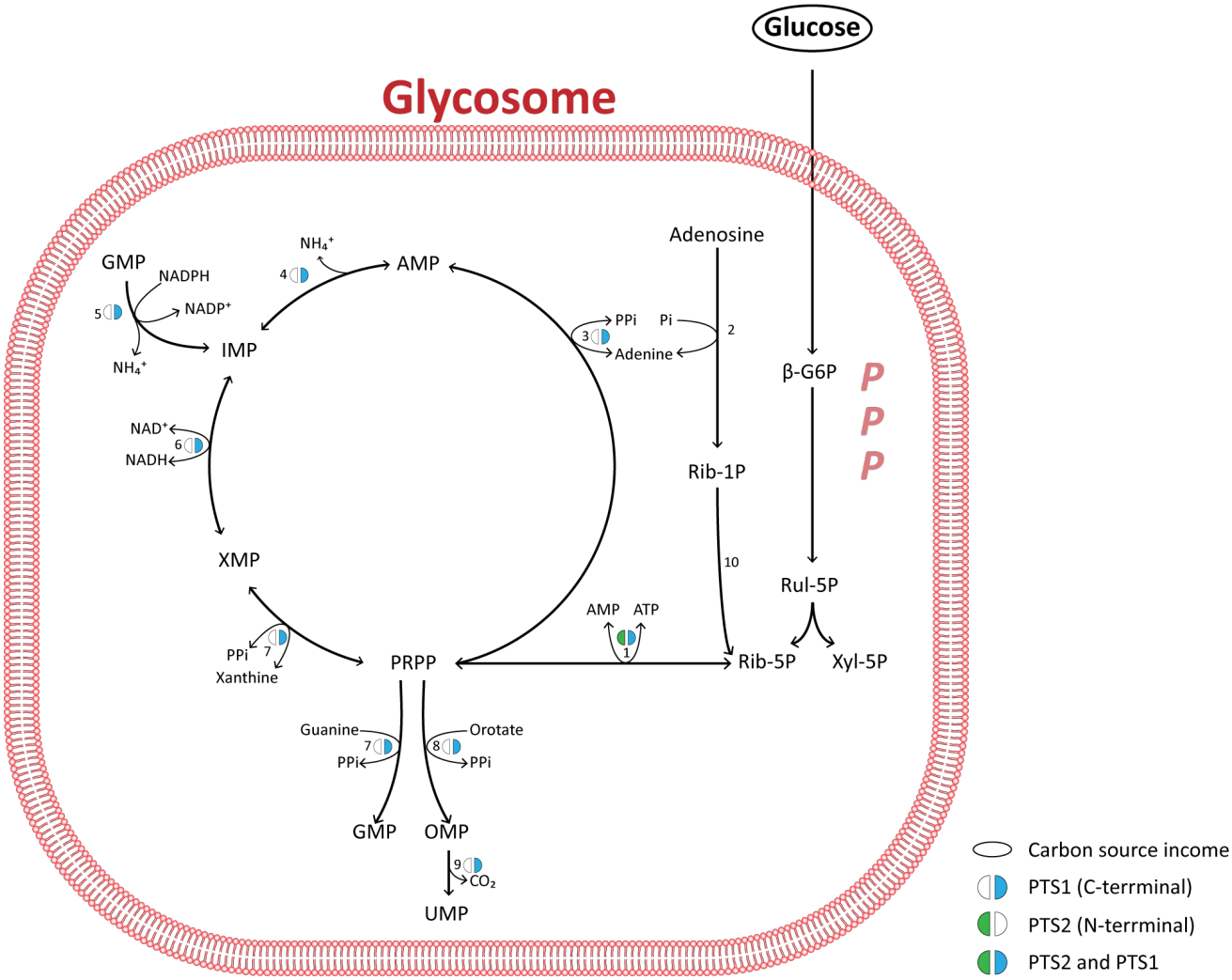

Supplementary Figure 3. Glycosomal pyrimidine biosynthesis and purine salvage. Enzymes: **1**. phosphoribosylpyrophosphate synthetase; **2.** nucleoside phosphorylase; **3**. adenine phosphoribosyltransferase; **4**. AMP deaminase; **5**. guanosine monophosphate reductase; **6**. inosine-5′-monophosphate dehydrogenase; **7**. hypoxanthine-guanine phosphoribosyltransferase; **8**. orotate phosphoribosyltransferase; **9**. orotidine-5-phosphate decarboxylase; **10**. phosphopentomutase. Metabolites: **Rul-5P**: ribulose 5-phosphate; **Rib-5P**: ribose 5-phosphate; **Xyl-5P**: xylulose 5-phosphate; **AMP**: adenosine monophosphate; **GMP**: guanosine monophosphate; **IMP**: inosine monophosphate; **OMP**: orotidine monophosphate; **PRPP:** phosphoribosyl-1-pyrophosphate; **UMP**: uridine monophosphate; **XMP**: xanthosine monophosphate. **PPP:** pentose-phosphate pathway.

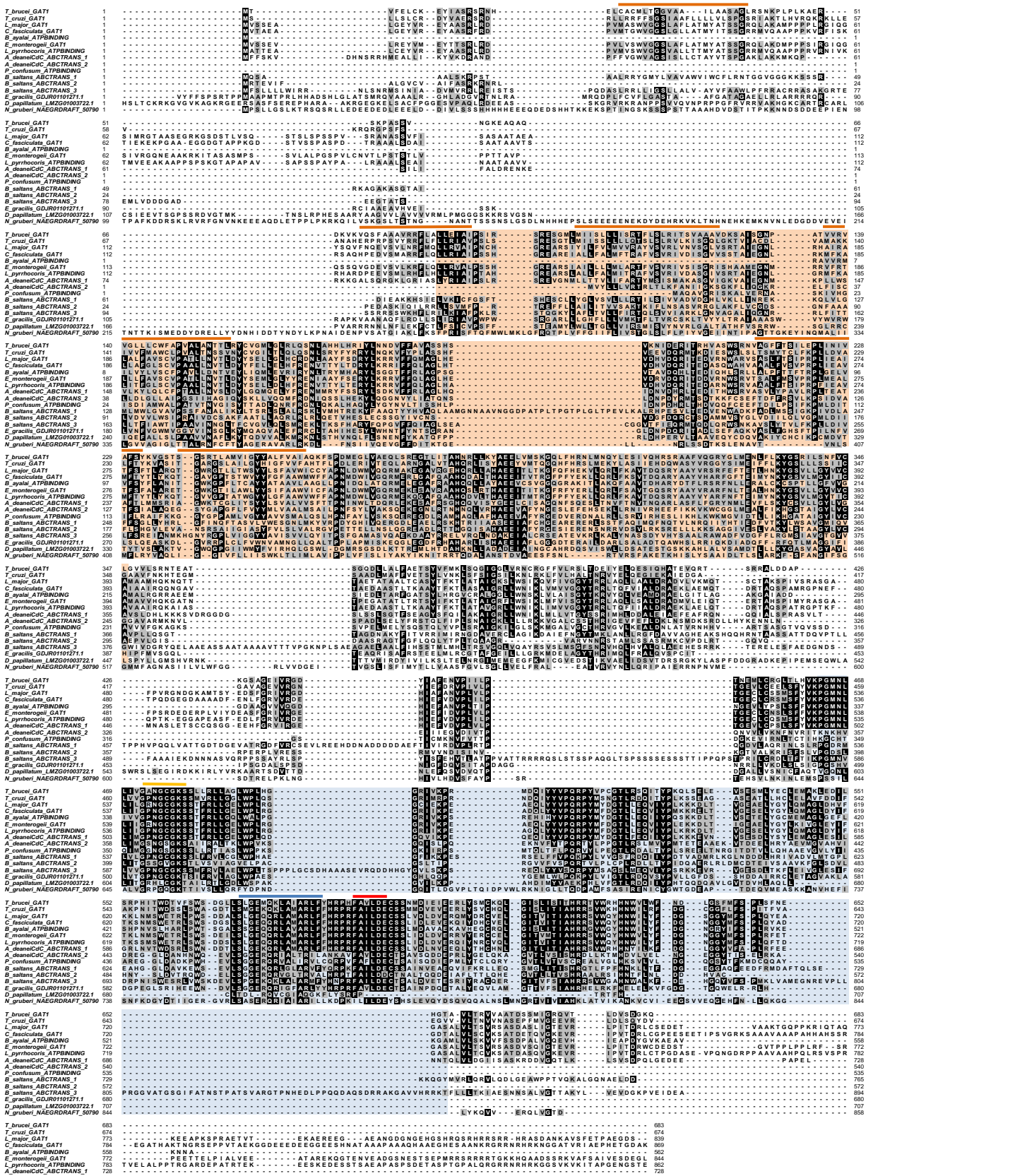

Supplementary Figure 4A. Alignment of Glycosomal ABC Transporter 1 (GAT1) amino-acid sequences from selected kinetoplastids and diplonemids, and homologous ABC transporters from *Euglena* and *Naegleria*. Sequence IDs: *T. brucei* GAT1: Tb927.4.4050; *T. cruzi*: TcCLB.508927.20; *L. major*: LmjF.31.0540; *C. fasciculata*: CFAC1_270013000; *B. ayalai*: Baya_280_0060*; E. monterogeii*: EMOLV88_310009900; *L. pyrrhocoris*: LpyrH10_32_0550; *A. deanei*: ADEAN_000145500, ADEAN_000310900; *P. confusum*: PCON_0002830; *B. saltans*: BSAL_11805, BSAL_54900, BSAL_84040; *E. gracilis*: GDJR01101271.1; *D. papillatum*: LMZG01003722.1; *N. gruberi*: NAEGRDRAFT_50790. The transmembrane domain (TMD) was predicted in InterProScan and is highlighted with an orange background. The transmembrane segments (TMSs) of the TMD are delineated by red lines above the alignment. The nucleotide-binding domain (NBD) is shown with a blue background. A conserved site ('ABC signature') is indicated with a blue line above the alignment. Yellow and red lines indicate the Walker A and B motifs, respectively. Domains and segments were specified by taking GAT1 of *T. brucei* as reference. The alignment was made with MAFFT https://www.ebi.ac.uk/Tools/msa/mafft/.

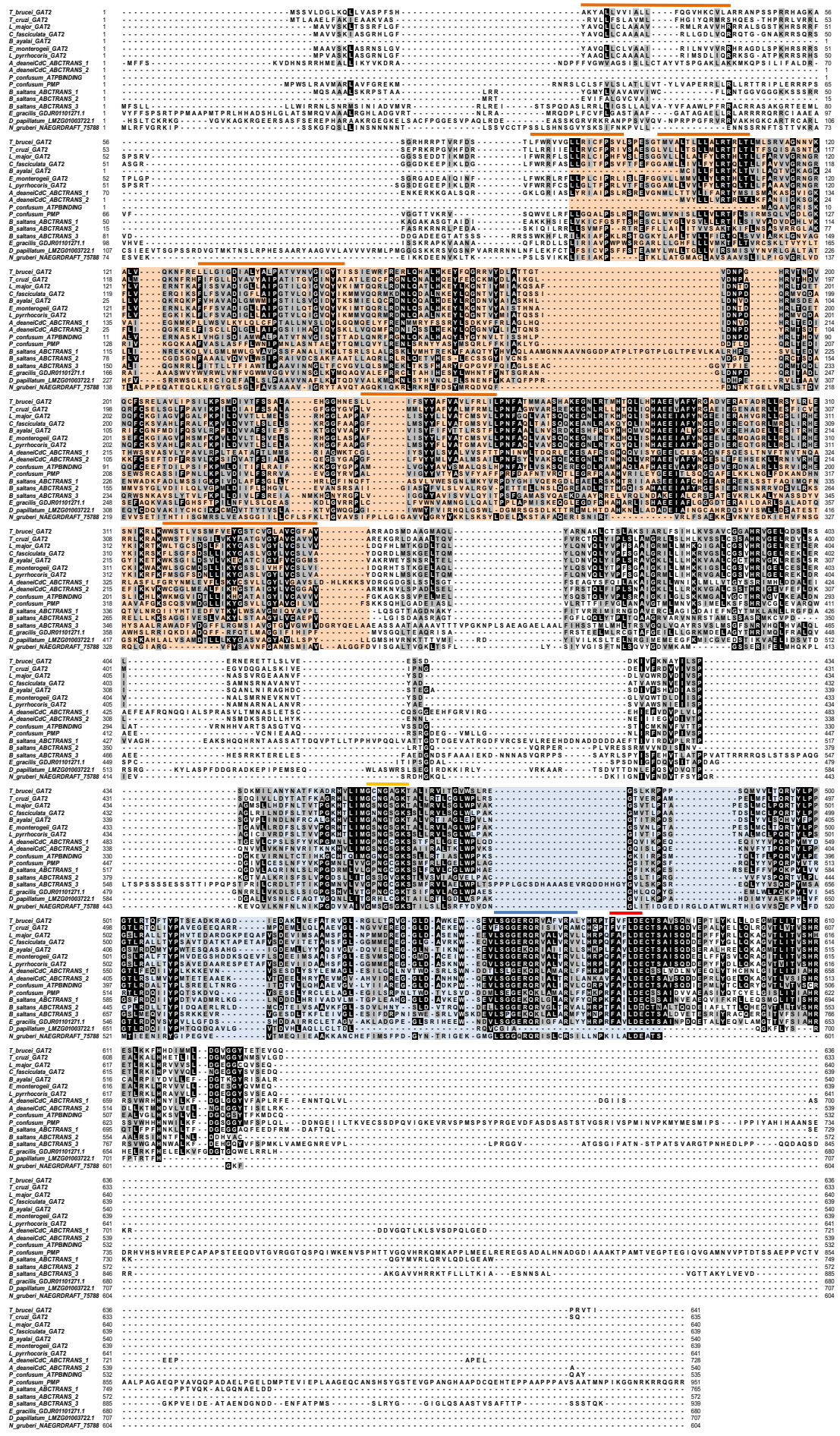

Supplementary Figure 4B. Alignment of Glycosomal ABC Transporter 2 (GAT2) amino-acid sequences from selected kinetoplastids and diplonemids, and homologous ABC transporters from *Euglena* and *Naegleria*. Sequence IDs: *T. brucei*: Tb927.11.3130; *T. cruzi*: TcCLB.510431.150; *L. major*: LmjF.33.1860; *C. fasciculata*: CFAC1_230022000; *B. ayalai*: Baya_003_0680; *E. monterogeii*: EMOLV88_330023900; *L. pyrrhocoris*: LpyrH10_07_2940; *A. deanei*: ADEAN_000145500, ADEAN_000310900; *P. confusum*: PCON_0002830, PCON_0030230; *B. saltans*: BSAL_11805, BSAL_54900, BSAL_84040; *E. gracilis*: GDJR01101271.1; *D. papillatum*: LMZG01003722.1; *N. gruberi*: NAEGRDRAFT_75788. The TMD was predicted with InterProScan and is highlighted with an orange background. The TMSs are delineated by red lines above the alignment. The NBD is shown with a blue background. A conserved site ('ABC signature') is indicated with a blue line above the alignment. The yellow and red lines indicate the Walker A and B motifs, respectively. Domains and segments were specified by taking GAT2 of *T. brucei* as reference. The alignment was made with MAFFT https://www.ebi.ac.uk/Tools/msa/mafft/.

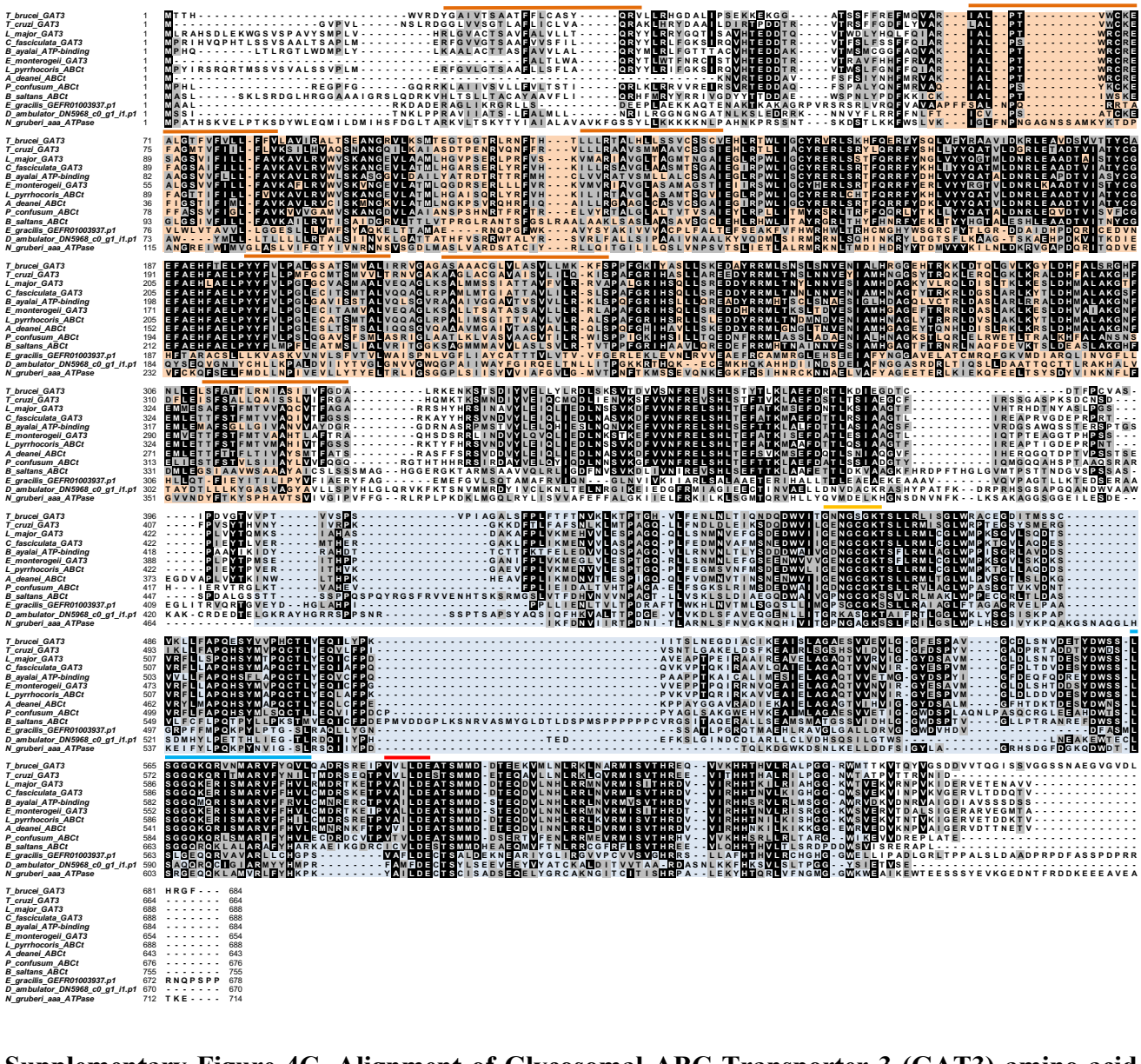

Supplementary Figure 4C. Alignment of Glycosomal ABC Transporter 3 (GAT3) amino-acid sequences from selected kinetoplastids and diplonemids, and homologous ABC transporters from *Euglena* and *Naegleria*. Sequence IDs: *T. brucei*: Tb927.11.1070; *T. cruzi*: TcCLB.506925.530; *L. major*: LmjF.27.0470; *C. fasciculata*: CFAC1_230048200; *B. ayalai*: Baya_102_0060; *E. monterogeii*: EMOLV88_270009200; *L. pyrrhocoris*: LpyrH10_35_0240; *A. deanei*: ADEAN_000284600; *P. confusum:* PCON_0004740; *B. saltans*: BSAL_4654; *E. gracilis*: GEFR01003937.p1; *D. ambulator*: DN5968_c0_g1_i1.p1; *N. gruberi*: NAEGRDRAFT_58406. The TMD is indicated by an orange background and the TMSs by orange lines above the alignment. The NBD is given with a blue background. Walker A and B motifs are indicated by yellow and red lines above the alignment, respectively. The blue line above the alignment shows the linker or conserved region ('ABC signature'). The indication of domains and important protein regions was made considering *T. cruzi* GAT3 as a reference. The alignment was made with MAFFT https://www.ebi.ac.uk/Tools/msa/mafft/.

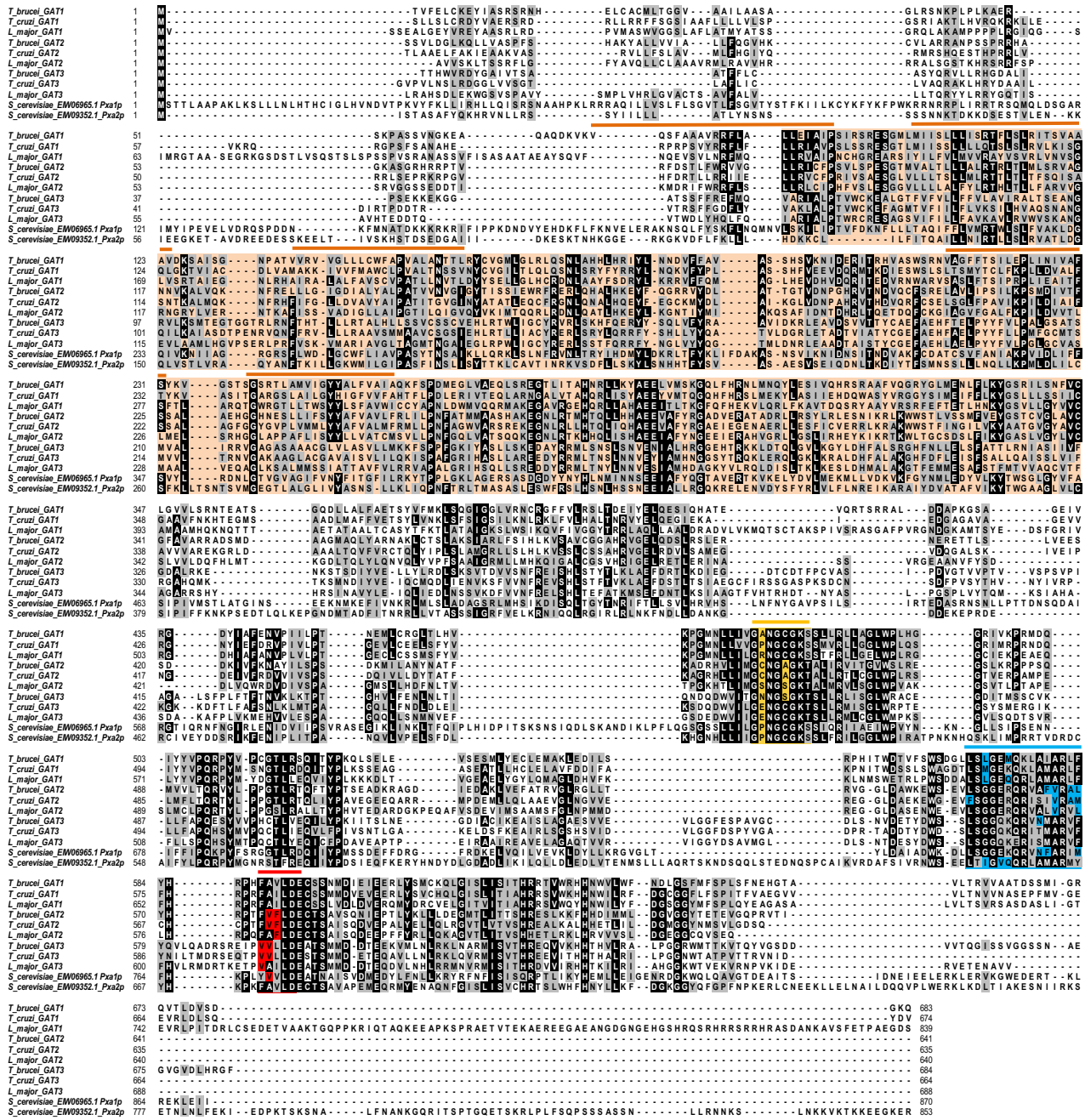

Supplementary Figure 4D. Alignment of the amino-acid sequences of the three Glycosomal ABC Transporters (GATs) from the TriTryps species and the two peroxisomal ABC transporters of *S. cerevisiae*. Sequence IDs: *T. brucei*: Tb927.4.4050 (GAT1), Tb927.11.3130 (GAT2), Tb927.11.1070 (GAT3); *T. cruzi:* TcCLB.508927.20 (GAT1), TcCLB.510431.150 (GAT2), TcCLB.506925.530 (GAT3); *L. major*: LmjF.31.0540 (GAT1), LmjF.33.1860 (GAT2), LmjF.27.0470 (GAT3); *S. cerevisiae:* EIW06965.1 (Pxa1p), EIW09352.1 (Pxa2p). The transmembrane domain TMD and the six transmembrane segments TMSs are indicated with an orange background and lines above the alignment, respectively. Indicated in yellow is the Walker A motif, in blue the linker or conserved site ('ABC signature') and in red the Walker B motif, according to Schneider and Hunke (1998). The alignment was made with MAFFT https://www.ebi.ac.uk/Tools/msa/mafft/.

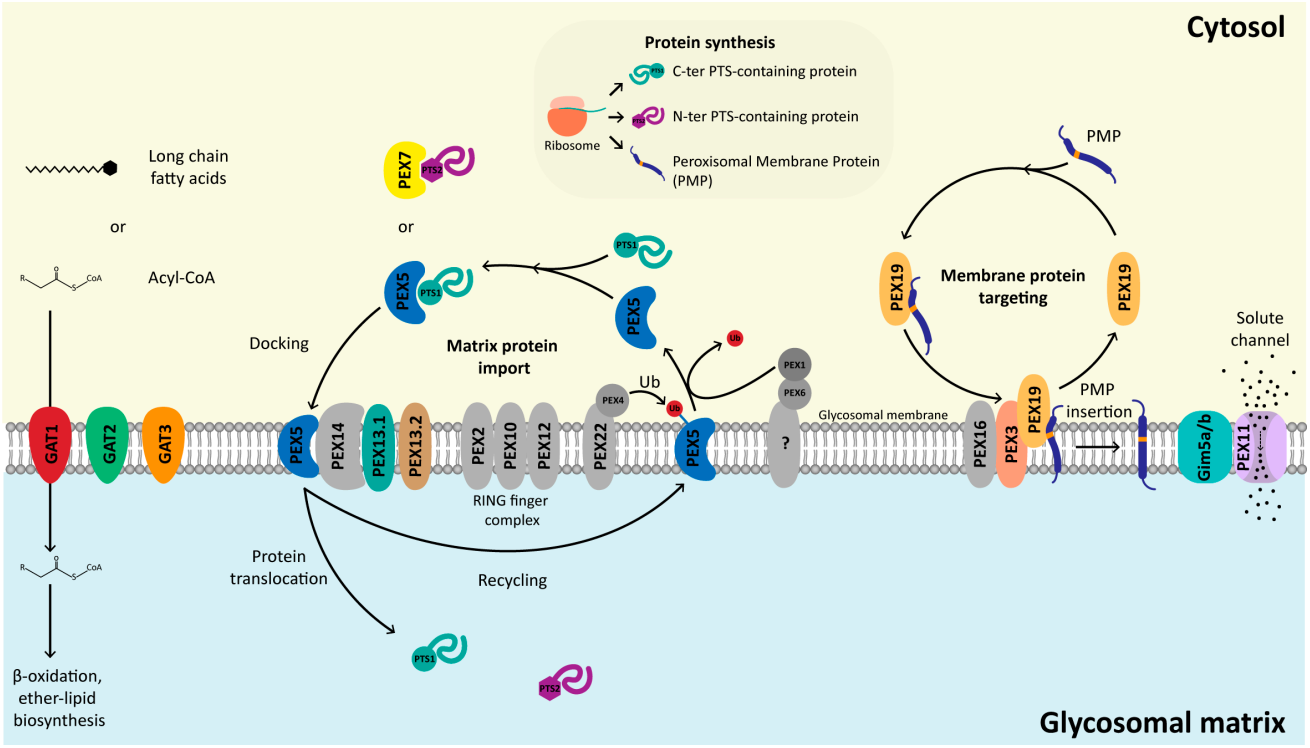

Supplementary Figure 5. Glycosome biogenesis in trypanosomes. Schematically indicated are several peroxins (PEXs), and their interactions resulting in the import of matrix proteins and insertion of membrane proteins. Peroxins involved in the successive stages of matrix protein import are: (i) receptors PEX5 and PEX7; (ii) docking complex components PEX13 and PEX14; (iii) PEX proteins involved in the ubiquitin-dependent retrieval of the receptors after having delivered the cargo in the matrix of the organelles: E3 ubiquitin ligases PEX2, PEX10 and PEX12, E2 ubiquitin-conjugation proteins PEX4 and its membrane anchor PEX22, and AAA-ATPases PEX1 and PEX6. Another set of peroxins is involved in insertion of membrane proteins: receptor and chaperone PEX19, docking protein PEX3, and import complex protein PEX16. Finally, PEX11, and its distant homologues GIM5A and GIM5A, are involved in proliferation of the organelles. Part of the processes shown are inferred from the results of studies performed on the biogenesis of mammalian and yeast peroxisomes. Only the peroxins represented in colour were considered and analysed in this work. **Ub**: Ubiquitin; **PMP**: Peroxisomal Membrane Protein: PEX1/6 anchor (PEX15, PEX26) were not detected in some members of Euglenozoa according to previous analysis (Jansen et al., 2021). Also shown are the three glycosomal ABC transporters (GATs), one of them (GAT1) responsible for import of acyl-CoAs/fatty acids (Igoillo-Esteve et al., 2011).

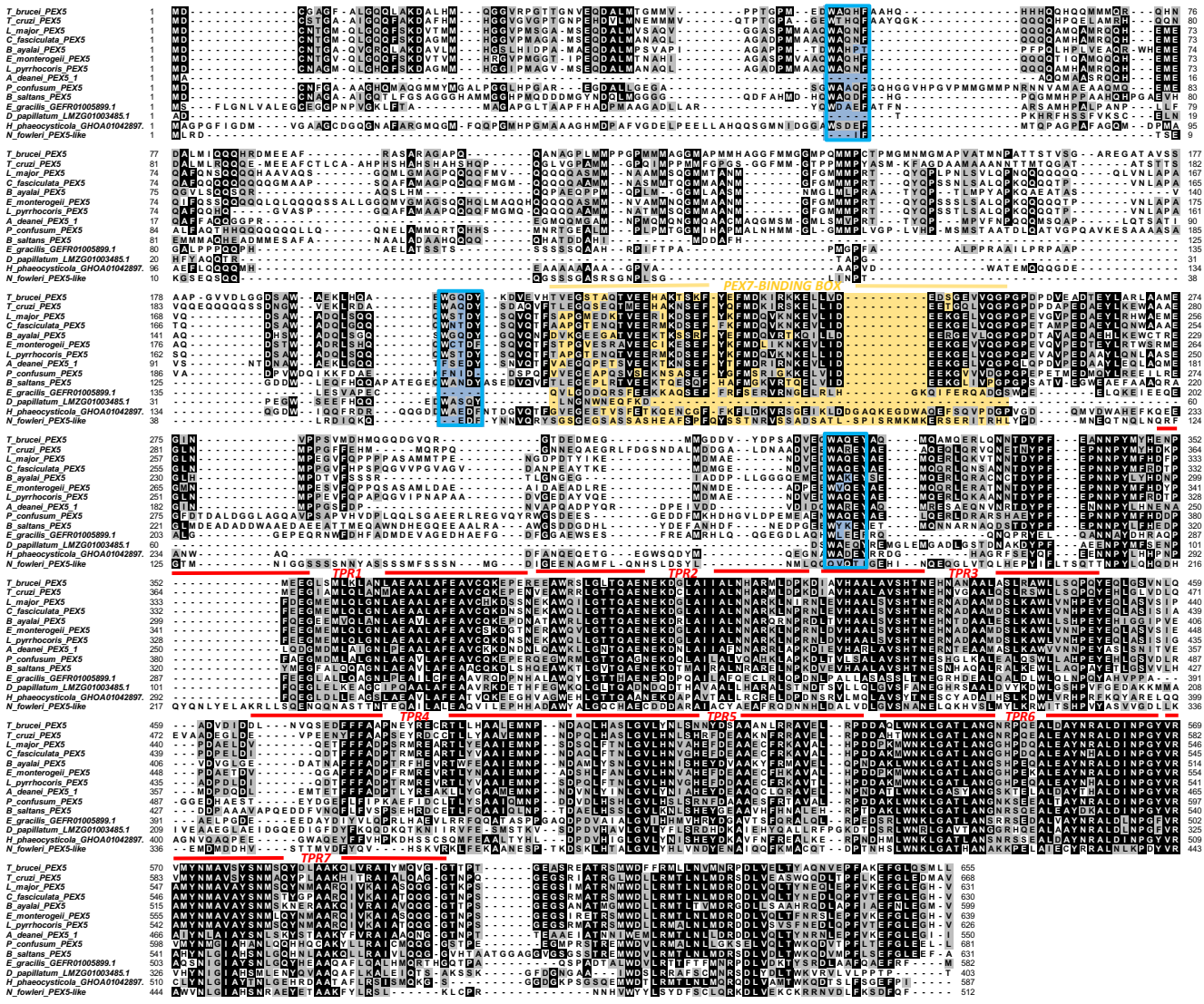

Supplementary Figure 6. Alignment of PEX5 amino-acid sequences from selected Euglenozoa and *Naegleria*. Sequence IDs: *T. brucei*: Tb927.5.1100; *T. cruzi*: TcCLB.511181.90; *L. major*: LmjF.35.1420; *C. fasciculata*: CFAC1_260016500; *B. ayalai*: Baya_285_0060; *E. monterogeii*: EMOLV88_350018200; *L. pyrrhocoris*: LpyrH10_01_3220; *A. deanei*: ADEAN_000139100; *P. confusum*: PCON_0019000; *B. saltans*: BSAL_07445; *E. gracilis*: GDJR01015915.1; *D. papillatum*: LMZG01003485.1; *H. phaeocysticola*: GHOA01042897.1; *N. fowleri*: NF0083570. Blue boxes in a blue background show the WxxxF/Y motifs (Choe et al., 2003), and the yellow line above the alignment the putative PEX7-binding box. The seven tetratricopeptide repeats (TPRs) are based on the *T. brucei* PEX5 sequence analysis by de Walque et al. (1999), and they are indicated with red lines above the alignment. The alignment was made with MAFFT https://www.ebi.ac.uk/Tools/msa/mafft/.

Previously, it has been reported that *T. brucei* PEX5 shows an overall sequence identity with homologues from human, yeasts and plants of 22-27%, with the C-terminal half better conserved (31-40%) than the N-terminal one (de Walque et al., 1999). PEX5 is quite well conserved among the Kinetoplastea with 40-90% identity between the different genera, decreasing to 20-30% when compared with other Euglenozoa. Within the Kinetoplastids, the highest conservation (74-90% identity is observed between the different Leishmaniinae genera (*Leishmania*, *Crithidia*, *Endotrypanum* and

Leptomonas) and somewhat less between *Trypanosoma* species (*T. brucei vs T. cruzi* 60%), a pattern also found for other peroxins (see below). Intriguingly, no WxxxF/Y pentapeptide motifs could be identified in the *N. fowleri* sequence that has been proposed as a PEX5 (González-Robles et al., 2020). The absence of such motifs and its mere 17-21% identity with the Euglenozoa sequences, raise the question how or if it can function as a PTS-protein import receptor.

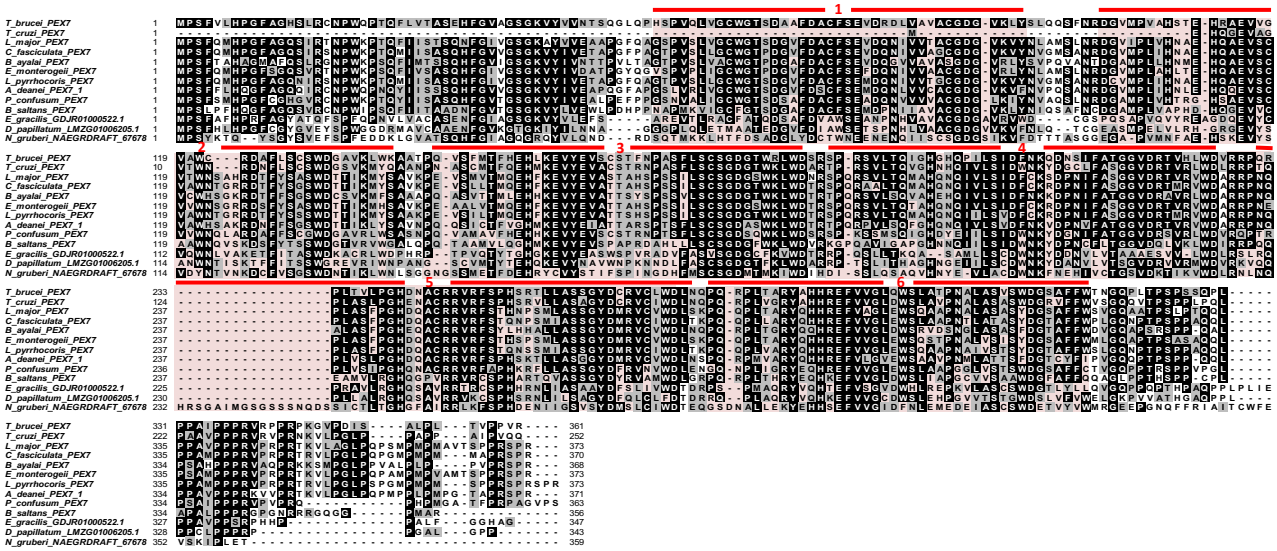

Supplementary Figure 7. Alignment of PEX7 amino-acid sequences from selected Euglenozoa and *Naegleria*. Sequence IDs: *T. brucei*: Tb927.3.3610; *T. cruzi*: TcCLB.503811.40; *L. major*: LmjF.29.0740; *C. fasciculata*: CFAC1_100019100; *B. ayalai*: Baya_199_0070*; E. monterogeii*: EMOLV88_360066500; *L. pyrrhocoris*: LpyrH10_08_0880; *A. deanei*: ADEAN_000081000*; P. confusum*: PCON_0043040; *B. saltans*: BSAL_21885; *D. papillatum*: LMZG01006205.1; *E. gracilis*: GDJR01000522.1; *N. gruberi*: NAEGRDRAFT_67678. The red lines above the alignment indicate the six WD40 repeats (Galland et al., 2007). The alignment was made with MAFFT https://www.ebi.ac.uk/Tools/msa/mafft/.

Previously, it has already been reported that the primary structure of the protein is well conserved: the TriTryps species are 65-92% identical to each other (81-92% between the Leishmaniinae genera, 49% between *T. brucei* and *T. cruzi*), and share about 30-35% identity with the human and *S. cerevisiae* PEX7s (Galland et al., 2007). In this work, we found approximately 50-60% identity between different genera of the Kinetoplastea, and still about 40% or higher when all Euglenozoa are compared. The PEX7s of euglenozoans and *N. gruberi* are 23-29% identical.

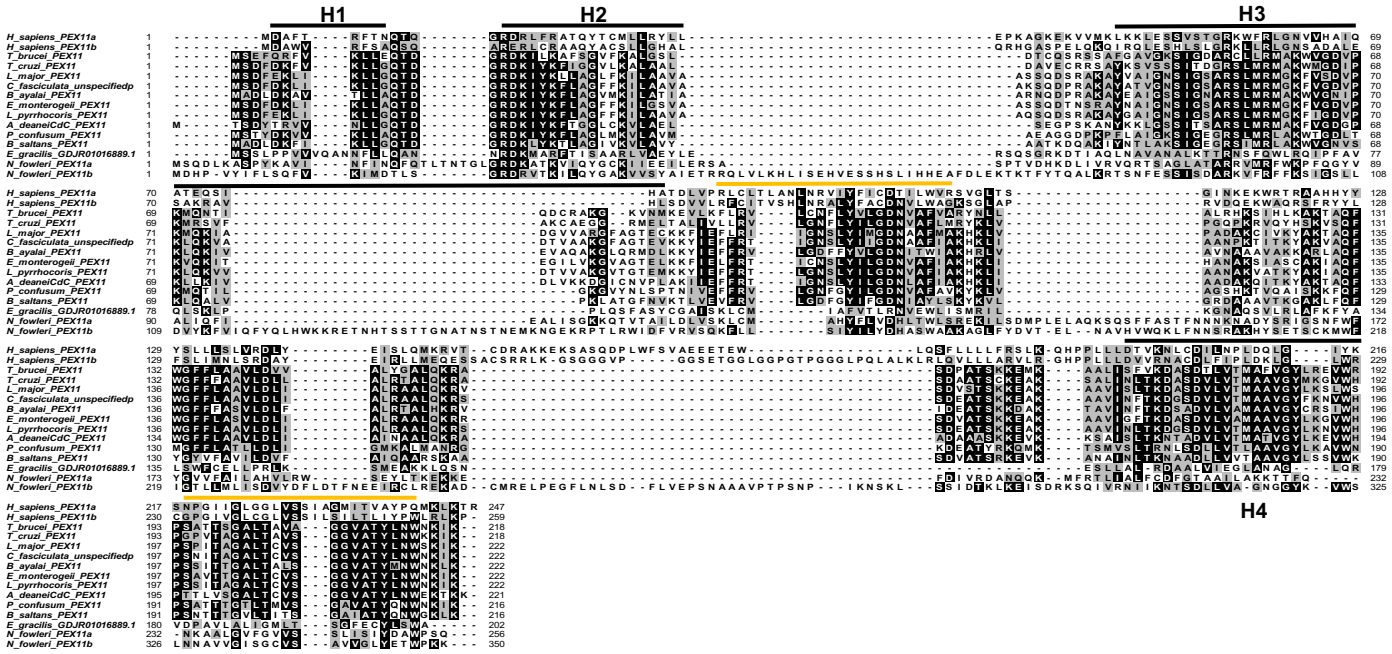

Supplementary Figure 8. Alignment of PEX11 amino-acid sequences from selected Euglenozoa, *Naegleria* and human. Sequence IDs: *H. sapiens*: O75192 (a), O96011 (b); *T. brucei*: Tb927.11.11520; *T. cruzi*: TcCLB.509203.40; *L. major*: LmjF.28.2260; *C. fasciculata*: CFAC1_300036700; *B. ayalai*: Baya_050_0050; *E. monterogeii*: EMOLV88_280024300; *L. pyrrhocoris*: LpyrH10_02_5310; *A. deanei*: ADEAN_000627600; *P. confusum*: PCON_0023900; *B. saltans*: BSAL_66630; *E. gracilis*: GDJR01016889.1; *N. fowleri*: NF0113850 (a), NF0009070 (b). The peptide regions forming three amphipathic helices (H1-H3) near the N-terminus and one near the C-terminus (H4) are indicated with black lines above the alignment (Opalinski et al., 2011; Schrader et al., 2016; Zientara-Rytter et al., 2022). The yellow lines show the predicted transmembrane regions (Lorenz et al., 1998; Schrader et al., 2012). The alignment was made with MAFFT https://www.ebi.ac.uk/Tools/msa/mafft/.

Conservation of PEX11s is low; while the human PEX11 isoforms exhibit about 40% identity between each other, and approximately 20% with *S. cerevisiae* PEX11 (Schrader et al., 1998), they have only 11-16% identity with the PEX11s from different Euglenozoa. Within the kinetoplastids the sequences are about 40-90% identical, with the highest conservation again between the Leishmaniinae genera, but they show only 12-14% identity with their *E. gracilis* and *N. fowleri* counterparts.

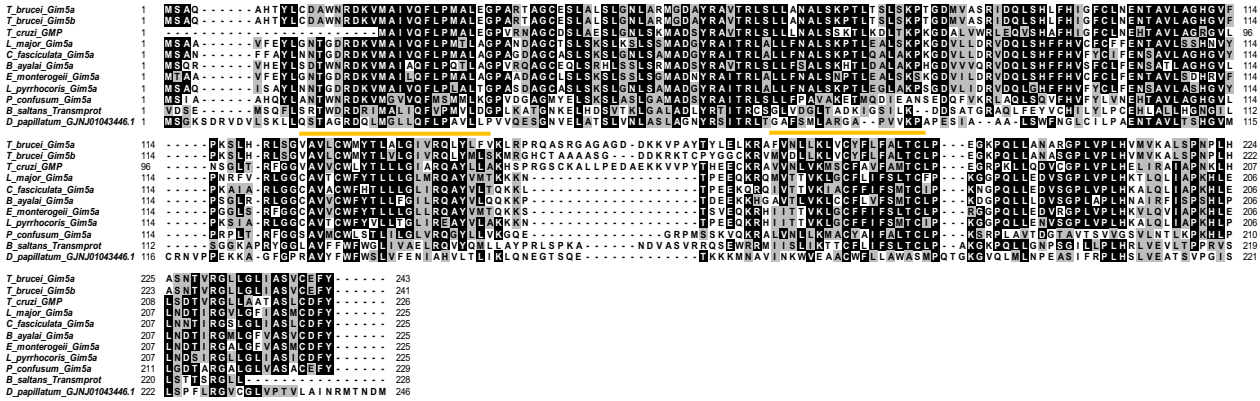

Supplementary Figure 9. Alignment of amino-acid sequences of GIM5A and GIM5B proteins from selected Euglenozoa. Sequence IDs: *T. brucei*: Tb927.9.11580, Tb927.9.11600; *T. cruzi*: TcCLB.507009.10; *L. major*: LmjF.35.3700; *C. fasciculata:* CFAC1_300087400; *B. ayalai:* Baya_100_0240; *E. monterogeii:* EMOLV88_350040400; *L. pyrrhocoris*: LpyrH10_01_6040; *P. confusum*: PCON_0000440; *B. saltans*: BSAL_50905; *D. papillatum*: GJNJ01043446.1. The yellow lines above the alignment indicate the transmembrane regions predicted in InterProScan and reported by Voncken et al. (2003). The alignment was made with MAFFT https://www.ebi.ac.uk/Tools/msa/mafft/.

The identity between GIM5 proteins of different kinetoplastids is 45-84%, and about 21-28% between kinetoplastid and *D. papillatum* GIM5.

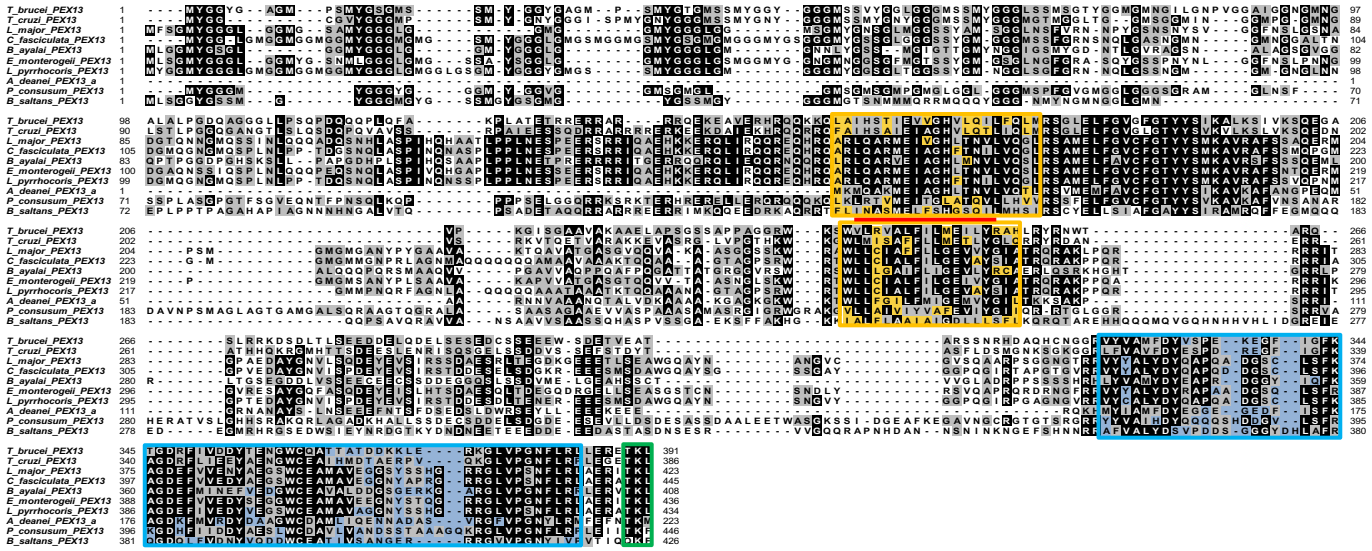

Supplementary Figure 10. Alignment of PEX13.1 amino-acid sequences from selected Kinetoplastea. Sequence IDs: *T. brucei*: Tb927.10.14720; *T. cruzi*: TcCLB.503833.30; *L. major*: LmjF.19.0070; *C. fasciculata*: CFAC1_170007100; *B. ayalai*: Baya_352_0040; *E. monterogeii*: EMOLV88_190005500; *A. deanei*: AGDE_11373 (a), AGDE_06786 (b); *L. pyrrhocoris*: LpyrH10_04_0070; *P. confusum*: PCON_0052590; *B. saltans*: BSAL_69630. The transmembrane regions, as predicted in InterProScan for the *T. brucei* amino-acid sequence, are indicated within the orange boxes, and the red line above the alignment shows the predicted PEX19-binding region (Verplaetse et al., 2009). The SH3 domain in the C-terminal part of the protein is highlighted by blue boundaries and background, while the C-terminal PTS1 motif is comprised in a green box. Identical residues are shown in black, and residues with similar physicochemical characteristics in grey. The alignment was made with MAFFT https://www.ebi.ac.uk/Tools/msa/mafft/.

PEX13.1 sequences detected in the databases for representatives of all kinetoplastid groups show considerable identities within groups of related taxa: 61-82% between different Leishmaniinae genera and 49% berween *T. brucei* and *T. cruzi*, but identities decrease to about 20-30% between distantly related trypanosomatids and bodonids. All kinetoplastid sequences possess the Tyr-Gly-rich N-terminal half found in peroxisomal PEX13s, but lack the upstream Pro-rich region, and they have two predicted transmembrane regions in the central part, and an SH3 (-like) domain in the C-terminal half followed by a PTS1.

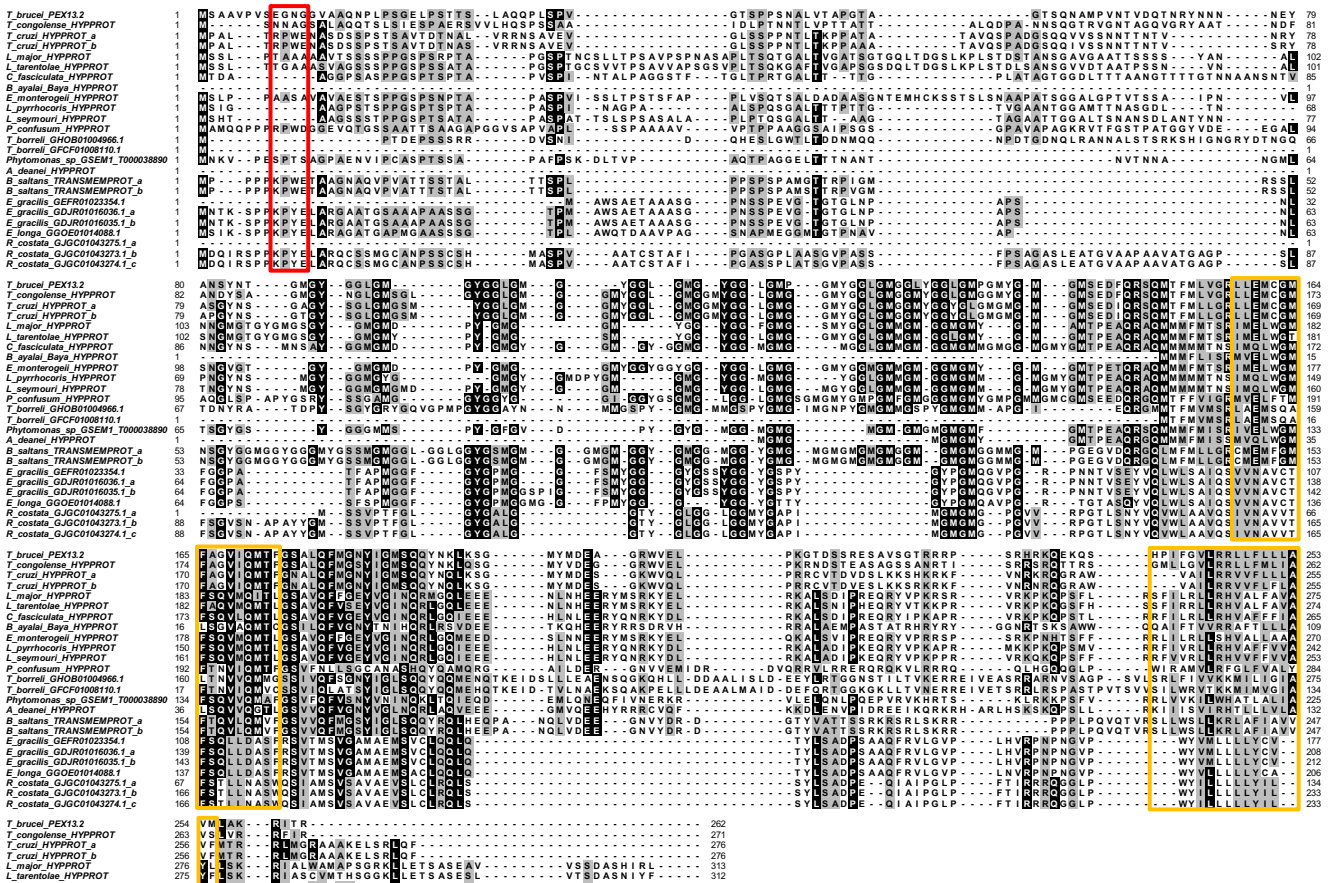

Supplementary Figure 11. **Alignment of PEX13.2 amino-acid sequences from selected Kinetoplastea**. Sequence IDs: *T. brucei*: Tb927.9.9430; *T. congolense*: TcIL3000_0_23750; *T. cruzi*: XM_813788.1 (a); XM_816431.1 (b); *L. major*: LmjF.35.4960; *L. tarentolae:* LtaP35.4940; *C. fasciculata:* CFAC1_300100900; *B. ayalai*: Baya_008_0560; *E. monterogeii*: EMOLV88_350051600; *L. pyrrhocoris*: XP_015665066.1; *L. seymouri*: KPI82997.1; *P. confusum*: PCON_0047390; *T. borreli*: GHOB01004966.1; GFCF01008110.1; *Phytomonas* sp.: GSEM1_T00003889001; A. deaneiCdC: ADEAN_000398700; *B. saltans*: BSAL_06635 (a); BSAL_06615 (b); *E. gracilis*: GEFR01023354.1; GDJR01016036.1 (a); GDJR01016035.1 (b); *E. longa*: GGOE01014088.1; *R. costata*: GJGC01043275.1 (a); GJGC01043273.1 (b); GJGC01043274.1 (c). The red box shows the K/R-P-W/Y-E/D motif present in some, but not all kinetoplastid PEX13.2s, and shared with many fungal, animal and plant PEX13s. The orange boxes indicate the transmembrane regions as predicted by Brennand et al. (2012). Sequences were retrieved from TriTrypDB and NCBI. The alignment was made with MAFFT https://www.ebi.ac.uk/Tools/msa/mafft/.

Potential PEX13.2 sequences were found for the kinetoplastids – trypanosomatids and bodonids – and euglenids (*E. gracilis*, *E. longa* and *R. costata*) analysed, but with a considerable a number of indels in

the sequences. Like for PEX13.1, important identity was found between *T. brucei* and *T. cruzi* (50%) and between Leishmaniinae genera (52-71%), but it dropped to about 10-25% between more distantly related kinetoplastids and with euglenids. Each of the sequences has a relatively high content of Pro residues in the N-terminal part, followed by a Tyr-Gly-rich region. Interestingly, most sequences, but not those of African trypanosomes, have a characteristic K/R-P-W/Y-E/D motif close to the N-terminus as previously already noticed (Brennand et al, 2012). This motif, whose function is not known yet, is shared with many fungal, animal and plant PEX13s, but not detected in the kinetoplastid PEX13.1s. Like previously reported for *T. brucei* PEX13.2, also all other sequences possess two predicted transmembrane regions and lack a SH3 domain.

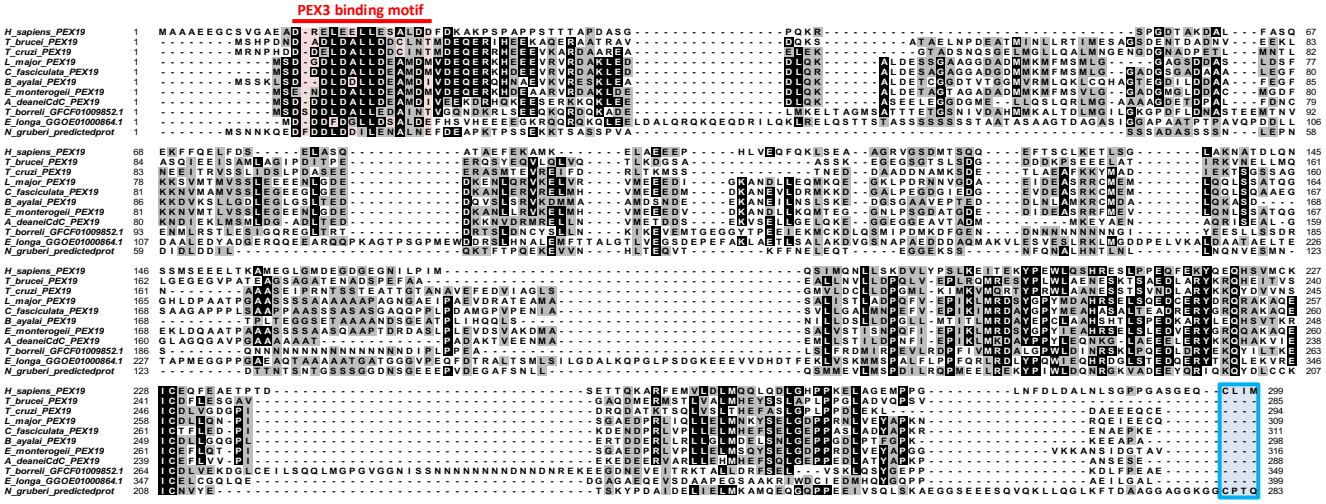

Supplementary Figure 12. Alignment of PEX19 amino-acid sequences from selected Euglenozoa, *Naegleria* and human. Sequence IDs: *H. sapiens*: NP_002848.1; *T. brucei*: Tb927.9.12290; *T. cruzi*: TcCLB.510737.100; *L. major*: LmjF.35.3260; *C. fasciculata*: CFAC1_300083000; *B. ayalai*: Baya_004_0210; *E. monterogeii*: EMOLV88_350036400; *A. deanei*: ADEAN_000416600; *T. borreli*: GFCF01009852.1; *E. longa*: GGOE01000864.1; *N. gruberi*: NAEGRDRAFT_75824. The PEX3 binding motif is indicated by the red line above the alignment and a red background (Kalel et al., 2019), while the CaaX motif for farnesylation, present at the C-terminus of only the *H. sapiens* and *N. gruberi* PEX19s, is shown with a blue background. The alignment was made in MUSCLE.

The (putative) PEX19 sequences detected in the databases for most kinetoplastids and some euglenids and *Naegleria* species, but not for diplonemids, show also considerable sequence variation: the identity between PEX19 from human and the Euglenozoa is about 10-16%, and 20% between human and *N. gruberi*. Values for PEX19s compared between different genera of kinetoplastids are 14% and 70%, with the highest identities (60-70%) found between the different Leishmaniinae genera. Importantly, the N-terminal PEX3 binding is well conserved in PEX19 of all species, whereas the C-terminal CaaX farnesylation motif is absent from the protein of all Euglenozoa but present in PEX19 of *Naegleria*.

References

Acosta, H., Burchmore, R., Naula, C., Gualdrón-López, M., Quintero-Troconis, E., Cáceres, A.J., Michels, P.A.M., Concepción, J.L., and Quiñones, W. (2019) Proteomic analysis of glycosomes from *Trypanosoma cruzi* epimastigotes. Mol. Biochem. Parasitol. 229, 62-74. doi: 10.1016/j.molbiopara.2019.02.008

Brennand, A., Rigden, D.J., and Michels, P.A.M (2012) Trypanosomes contain two highly different isoforms of peroxin PEX13 involved in glycosome biogenesis. FEBS Lett. 586(13), 1765-1771. doi: 10.1016/j.febslet.2012.05.019

Choe, J., Moyersoen, J., Roach, C., Carter, T.L., Fan, E., Michels, P.A.M., and Hol WG.J. (2003) Analysis of the sequence motifs responsible for the interactions of peroxins 14 and 5, which are involved in glycosome biogenesis in *Trypanosoma brucei.* Biochemistry. 42(37), 10915-10922. doi: 10.1021/bi034248n

Colasante, C., Ellis, M., Ruppert, T., and Voncken, F. (2006) Comparative proteomics of glycosomes from bloodstream form and procyclic culture form *Trypanosoma brucei brucei.* Proteomics. 6(11), 3275-3293. doi: 10.1002/pmic.200500668

Colasante, C., Voncken, F., Manful, T., Ruppert, T., Tielens, A.G.M., Van Hellemond, J.J., and Clayton, C. (2013) Proteins and lipids of glycosomal membranes from *Leishmania tarentolae* and *Trypanosoma brucei.* F1000Res. 2:27. doi: 10.12688/f1000research.2-27.v1

de Walque, S., Kiel, J.A.K.W., Veenhuis, M., Opperdoes, F.R., and Michels, P.A.M. (1999) Cloning and analysis of the PTS-1 receptor in *Trypanosoma brucei.* Mol. Biochem. Parasitol. 104(1), 106-119. doi: 10.1016/s0166-6851(99)00144-9

Galland, N., Demeure, F., Hannaert, V., Verplaetse, E., Vertommen, D., Van der Smissen, P., Courtoy P.J., and Michels, P.A.M. (2007) Characterization of the role of the receptors PEX5 and PEX7 in the import of proteins into glycosomes of *Trypanosoma brucei.* Biochim. Biophys. Acta. 1773, 521-535. doi:10.1016/j.bbamcr.2007.01.006

González-Robles, A., González-Lázaro, M., Lagunes-Guillén, A.E., Omaña-Molina, M., Lares-Jiménez, L.F., Lares-Villa, F., and Martínez-Palomo, A. (2020) Ultrastructural, Cytochemical, and Comparative Genomic Evidence of Peroxisomes in Three Genera of Pathogenic Free-Living Amoebae, Including the First Morphological Data for the Presence of This Organelle in Heteroloboseans. Genome Biol. Evol. 12(10), 1734-1750. doi: 10.1093/gbe/evaa129

Gualdrón-López, M., Vapola, M.H., Miinalainen, I.J., Hiltunen, J.K., Michels, P.A.M., and Antonenkov, V.D. (2012) Channel-forming activities in the glycosomal fraction from the bloodstream form of *Trypanosoma brucei.* PLoS One. 7(4):e34530. doi: 10.1371/journal.pone.0034530

Güther, M.L., Urbaniak, M.D., Tavendale, A., Prescott, A., and Ferguson, M.A. (2014) High-confidence glycosome proteome for procyclic form *Trypanosoma brucei* by epitope-tag organelle enrichment and SILAC proteomics. J. Proteome Res. 13(6), 2796-2806. doi: 10.1021/pr401209w

Igoillo-Esteve, M., Mazet, M., Deumer, G., Wallemacq, P., and Michels, P.A.M. (2011) Glycosomal ABC transporters of *Trypanosoma brucei*: characterisation of their expression, topology and substrate specificity. Int. J. Parasitol. 41(3-4), 429-438. doi: 10.1016/j.ijpara.2010.11.002

Jansen, R.L.M., Santana-Molina, C., Van den Noort, M., Devos, D.P., and Van der Klei, I.J. (2021) Comparative Genomics of Peroxisome Biogenesis Proteins: Making Sense of the PEX Proteins. Front. Cell. Dev. Biol. 9:654163. doi: 10.3389/fcell.2021.654163

Kalel, V.C., Li, M., Gaussmann, S., Delhommel, F., Schäfer, A.B., Tippler, B., Jung, M., Maier, R., Oeljeklaus, S., Schliebs, W., Warscheid, B., Sattler, M., and Erdmann, R. (2019) Evolutionary divergent PEX3 is essential for glycosome biogenesis and survival of trypanosomatid parasites. Biochim. Biophys. Acta Mol. Cell Res. 1866(12):118520. doi: 10.1016/j.bbamcr.2019.07.015

Leroux, A.E., and Krauth-Siegel, R.L. (2016) Thiol redox biology of trypanosomatids and potential targets for chemotherapy. Mol. Biochem. Parasitol. 206(1-2), 67-74. doi: 10.1016/j.molbiopara.2015.11.003

Lorenz, P., Maier, A.G., Baumgart, E., Erdmann, R., and Clayton, C. (1998) Elongation and clustering of glycosomes in *Trypanosoma brucei* overexpressing the glycosomal Pex11p. EMBO J. 17, 3542-3555. doi:10.1093/emboj/17.13.3542

Michels P.A.M., and Gualdrón-López, M. (2022) Biogenesis and metabolic homeostasis of trypanosomatid glycosomes: new insights and new questions. J. Eukaryot. Microbiol., accepted for publication. doi: 10.1111/jeu.12897

Naderer, T., Heng, J., and McConville, M.J. (2010) Evidence that intracellular stages of *Leishmania major* utilize amino sugars as a major carbon source. PLoS Pathog. 6(12):e1001245. doi: 10.1371/journal.ppat.1001245

Opaliński, Ł., Kiel, J.A., Williams, C., Veenhuis, M., and Van der Klei, I.J. (2011) Membrane curvature during peroxisome fission requires Pex11. EMBO J. 30(1), 5-16. doi: 10.1038/emboj.2010.299

Quiñones, W., Acosta, H., Gonçalves, C.S., Motta, M.C.M., Gualdrón-López, M., and Michels, P.A.M. (2020) Structure, Properties, and Function of Glycosomes in *Trypanosoma cruzi.* Front. Cell. Infect. Microbiol. 10:25. doi: 10.3389/fcimb.2020.00025

Schneider, E., and Hunke, S. (1998) ATP-binding-cassette (ABC) transport systems: functional and structural aspects of the ATP-hydrolyzing subunits/domains. FEMS Microbiol. Rev. 22(1), 1-20. doi: 10.1111/j.1574-6976.1998.tb00358.x

Schrader, M., Reuber, B.E., Morrell, J.C., Jimenez-Sanchez, G., Obie, C., Stroh, T.A., Valle, D., Schroer, T.A., and Gould, S.J. (1998) Expression of PEX11beta mediates peroxisome proliferation in the absence of extracellular stimuli. J. Biol. Chem. 273(45), 29607-29614. doi: 10.1074/jbc.273.45.29607

Schrader, M., Bonekamp, N.A., and Islinger, M. (2012) Fission and proliferation of peroxisomes. Biochim. Biophys. Acta. 1822(9), 1343-1357. doi: 10.1016/j.bbadis.2011.12.014

Schrader, M., Costello, J.L., Godinho, L.F., Azadi, A.S., and Islinger, M. (2016) Proliferation and fission of peroxisomes - An update. Biochim. Biophys. Acta. 1863, 971-983. doi:10.1016/j.bbamcr.2015.09.024

Verplaetse, E., Rigden, D.J., and Michels, P.A.M. (2009) Identification, characterization and essentiality of the unusual peroxin 13 from *Trypanosoma brucei.* Biochim. Biophys. Acta. 1793(3), 516-527. doi: 10.1016/j.bbamcr.2008.12.020

Vertommen, D., Van Roy, J., Szikora, J.P., Rider, M.H., Michels, P.A.M., and Opperdoes, F.R. (2008) Differential expression of glycosomal and mitochondrial proteins in the two major life-cycle stages of *Trypanosoma brucei.* Mol. Biochem. Parasitol. 158(2), 189-201. doi: 10.1016/j.molbiopara.2007.12.008

Voncken, F., Van Hellemond, J.J., Pfisterer, I., Maier, A., Hillmer, S., and Clayton, C. (2003) Depletion of GIM5 causes cellular fragility, a decreased glycosome number, and reduced levels of ether-linked phospholipids in trypanosomes. J. Biol. Chem. 278, 35299-35310. doi:10.1074/jbc.M301811200

Zientara-Rytter, K.M., Mahalingam, S.S, Farré, J.C., Carolino, K., and Subramani, S. (2022) Recognition and Chaperoning by Pex19, Followed by Trafficking and Membrane Insertion of the Peroxisome Proliferation Protein, Pex11. Cells. 11(1):157. doi: 10.3390/cells11010157
